# A minimal transcription factor network is sufficient to drive paclitaxel biosynthesis

**DOI:** 10.1126/sciadv.aee6211

**Published:** 2026-06-05

**Authors:** Qiaona Pan, Marisol Ochoa-Villarreal, Susan Howat, Rabia Amir, Zejun Yan, Christopher E. French, Gary J. Loake, Beimi Cui

**Affiliations:** ^1^ School of Horticulture and Landscape Architecture, Yangzhou University, Yangzhou, Jiangsu 225009, China.; ^2^ Institute of Molecular Plant Sciences, School of Biological Sciences, University of Edinburgh, Edinburgh EH9 3BF, UK.; ^3^ Atta-ur-Rahman School of Applied Biosciences (ASAB), National University of Sciences & Technology (NUST), Islamabad 44000, Pakistan.; ^4^ Centre for Engineering Biology, School of Biological Sciences, University of Edinburgh, Edinburgh EH9 3BF, UK.

## Abstract

The supply of the anticancer drug paclitaxel is limited by its low natural abundance and complex chemical structure. We previously reported the development of a cultured *Taxus* cambial meristematic cell (CMC) system, where methyl jasmonate enhances paclitaxel production. In this report, we describe a comprehensive analysis to identify potential master regulators of paclitaxel biosynthesis. We report that the combined expression of two myeloblastosis transcription factors can cooperatively activate a subset of paclitaxel biosynthetic genes in stable transgenic CMCs, resulting in a 121-fold increase in paclitaxel and a 364-fold increase in its valuable precursor baccatin III. Our findings provide a powerful approach to enhancing production of this vital compound and can also be applied to other valuable products.

## Teaser

Bioengineering *Taxus* cells with two MYB TFs greatly boosts yields of the anticancer drug paclitaxel and its precursor.

## INTRODUCTION

Paclitaxel (marketed as Taxol) is a diterpenoid natural product that is a blockbuster anticancer drug. Since its initial Food and Drug Administration (FDA) approval for ovarian cancer in 1992, its application has expanded to include the treatment of breast, pancreatic and non–small cell lung cancer (*1*, *2*). Paclitaxel’s potent activity is derived from its complex molecular architecture, which is assembled through an elaborate biosynthetic pathway comprising over 19 enzymatic steps starting from geranylgeranyl diphosphate (*3*–*5*). Current commercial supplies of paclitaxel rely on a costly and resource-intensive semisynthesis process, which starts with intermediates, such as baccatin III, that are extracted from *Taxus* trees, and on bioreactor-based plant cell culture processes (*3*, *6*). Consequently, the global demand for this World Health Organization–essential medicine consistently outstrips its supply, underscoring the urgent need for a more sustainable and scalable production platform.

Engineered cell culture has emerged as a promising strategy for producing paclitaxel in a more environmentally friendly manner (*7*–*9*). Notable progress has been made at producing early paclitaxel precursors in microbial systems, such as *Escherichia coli* and yeast (*10*, *11*), but their efficacy is limited because of challenges in expressing essential cytochrome P450 enzymes (*12*). In contrast, plant platforms provide a superior environment for expressing cytochrome P450–dependent pathways, owing to their native complement of redox partners and subcellular compartments (*13*, *14*). Recent research has successfully identified the key missing enzymes in paclitaxel biosynthesis, such as T2′OGD (2′α hydroxylation), T3′NBT (3′-N benzoylation) (*4*), taxane oxetanase 1 (*15*), and facilitator of taxane oxidation 1 (FoTO1) (*5*). This has enabled the successful reconstitution of the biosynthetic pathway to baccatin III and even paclitaxel in heterologous hosts (*11*, *12*, *15*–*18*). By integrating genomics and synthetic biology to optimize gene expression, multiple groups have successfully produced the key intermediate baccatin III in *Nicotiana benthamiana*, at yields ranging from ~50 ng/g to 10 to 30 μg/g in reconstituted pathways (*5*, *15*, *16*, *18*). A recent study showed that a minimal gene set expressed in *N. benthamiana* was able to produce baccatin III at levels approaching those observed in *Taxus* needles (*18*). Although these heterologous systems provide a promising platform for sustainable production, the resulting titers of paclitaxel and baccatin III remain low for industrial application (*19*).

We previously reported the development of a cultured *Taxus* CMC (cambial meristematic cell) system derived from vascular stem cells (*20*) and the ability of Methyl Jasmonate (MeJA) elicitation to significantly increase paclitaxel production in this system. Our aim was to further improve the production of paclitaxel in this system by identifying potential master regulators of paclitaxel biosynthesis. Here, we report the identification and validation of two myeloblastosis (MYB) transcription factors (TFs) that can significantly increase paclitaxel production in CMCs. Stable transgenic *Taxus* CMCs expressing these two MYBs were able to significantly enhance paclitaxel, reaching 98 μg/g fresh weight (FW), about 121 times greater compared to control cells, and the key intermediate baccatin III, reaching 877 μg/g FW, about 364 times the level seen in the control. We present a TF engineering approach that not only elucidates the hierarchical control of this complex biosynthetic pathway but also achieves promising titers for industrial translation.

## RESULTS

### Identification of TFs that are up-regulated after MeJA elicitation

**Fig. 1.**
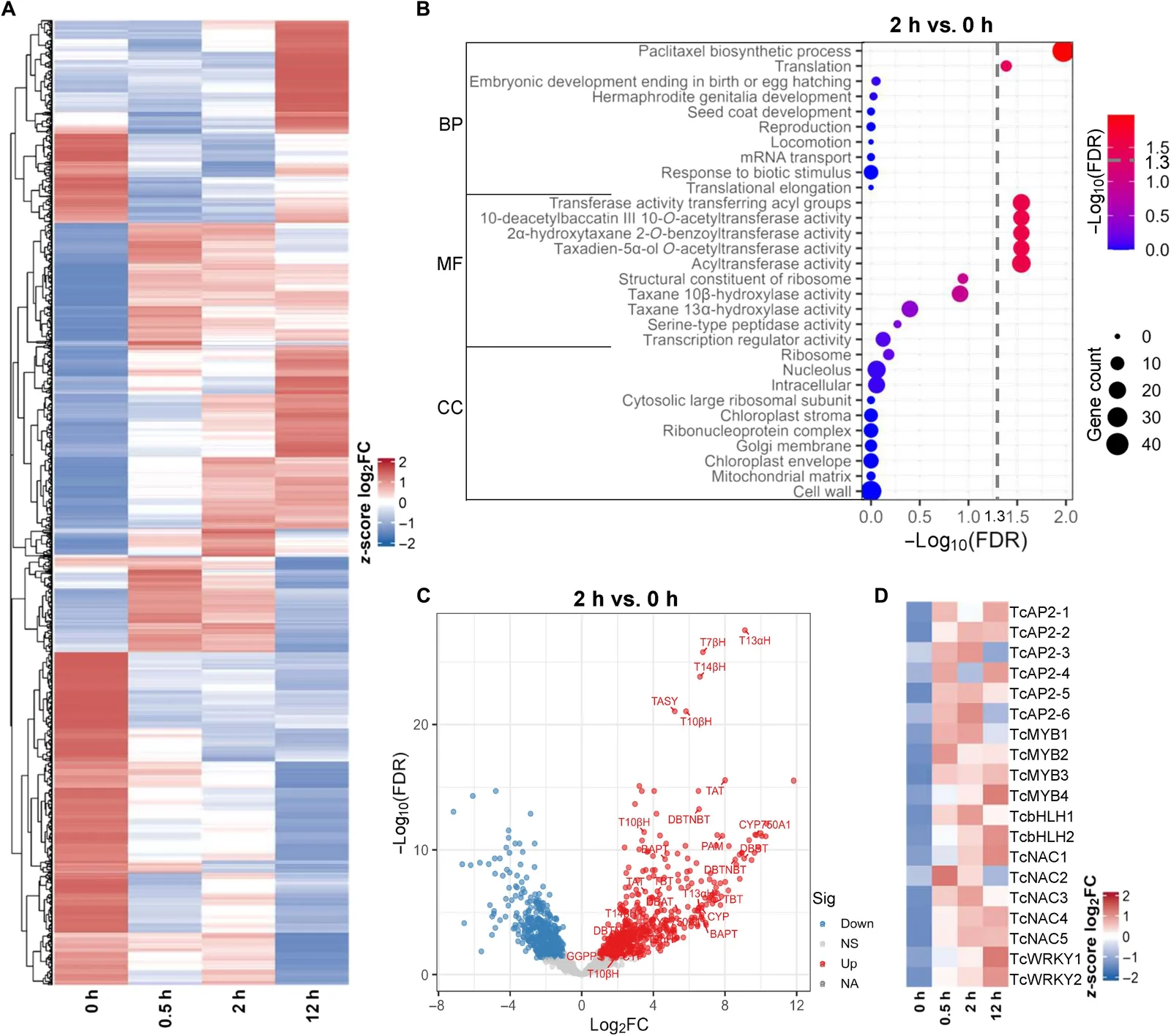
Transcriptomic changes in CMC cells after MeJA treatment reveal potential TFs. (**A**) Heatmap of *Taxus* CMCs upon 100 μM MeJA treatment at 0.5, 2, and 12 hours (h). Values represent *z*-score–normalized log_2_ fold changes (log_2_FCs) of significant DEGs. Rows correspond to genes, and columns correspond to time points. Color scale ranges from blue (down-regulated) to red (up-regulated). (**B**) Dot plots showing the top 10 enriched GO categories for Biological Process (BP), Molecular Function (MF), and Cellular Component (CC) terms among DEGs at 2 hours after MeJA treatment compared to 0 hours. Circle size indicates the number of DEGs per term, and color intensity reflects statistical significance [−log_10_ false discovery rate (FDR)]. The dashed line indicates –log_10_ FDR = 1.3 (FDR = 0.05). (**C**) Volcano plot showing differential gene expression in CMCs after 2 hours of MeJA treatment (|log_2_FC| ≥ 1, FDR < 0.05; red, up-regulated; blue, down-regulated; white, NS, not significant; gray, NA, not available). Key paclitaxel-responsive enzyme coding genes are labeled. Differential expression was determined using DESeq2 (*n* = 3). (**D**) Heatmap showing expression profiles of identified TFs across MeJA treatment time points (0.5, 2, and 12 hours).

The transcriptional cascade triggered in *Taxus cuspidata* (*Tc*) CMCs after MeJA elicitation was observed, during which paclitaxel production was significantly induced (*20*, *21*). To identify TFs for bioengineering *Taxus* cell, we treated CMCs with MeJA, and this elicitation induced paclitaxel and baccatin III (fig. S1A), consistent with previous findings (*20*, *21*). Transcriptome analysis showed that MeJA triggered an early (0.5 hours) mostly inductive wave in gene expression in CMCs, followed by a second later wave (12 hours) of genes that are up-and down-regulated, 40 and 60%, respectively (Fig. 1A). Transcriptomic profiling identified 867 up-regulated and 749 down-regulated genes following elicitation with MeJA (fig. S1, B and C). We performed a gene ontology (GO) enrichment analysis on differentially expressed genes (DEGs), and the results showed a significant enrichment of DEGs in the paclitaxel biosynthetic process and genes encoding various taxane-related enzyme activities, such as 10-deacetylbaccatin III 10-*O*-acetyltransferase (Fig. 1B and fig. S1D). This strong enrichment demonstrates that MeJA treatment activates the core molecular network responsible for paclitaxel biosynthesis. Consistent with these data, the volcano plot shows that the treatment at 2 hours led to a massive and highly significant up-regulation of genes in the paclitaxel biosynthetic pathway (Fig. 1C). As highlighted in Fig. 1C, key genes encoding early- and late-stage enzymes (fig. S1E) such as taxadiene synthase (TASY), taxadiene-13α-hydrolase (T13αH), taxane-10β-hydrolase (T10βH), and taxane-2α-ol-*O*-benzoyl transferase (DBBT) are among the most highly and significantly up-regulated genes. This provides compelling evidence that MeJA treatment directly activates the expression of the core enzyme-coding genes required for paclitaxel production.

From the 1646 DEGs, we identified 79 TFs spanning 19 different gene families (fig. S2A). The APETALA2 (AP2) family was the largest, followed by MYB, basic helix-loop-helix (bHLH), C3H zinc finger, and NAM, ATAF1,2, and CUC2 (NAC) families. Our analysis showed that MeJA treatment up-regulated 50 of these TFs, with a high proportion belonging to the MYB, C3H, NAC, and WRKY families. Conversely, 29 TFs were down-regulated, prominently featuring members of the AP2, basic leucine zipper, and CCAAT TF families (fig. S2A). To identify potential positive regulators of paclitaxel biosynthesis, we focused on TFs that were rapidly and significantly up-regulated at 0.5 hours post–MeJA treatment. This selection yielded 19 candidate TFs, comprising six AP2, four MYB, five NAC, two bHLH, and two WRKY proteins (Fig. 1D). These TFs exhibited temporal separation in gene expression, suggesting potentially distinct roles in transcriptional regulation (Fig. 1D and fig. S2B). For instance, TcAP2-5 and TcMYB3 were highly induced at 0.5 hours, whereas TcMYB4, TcNAC1, TcWRKY1, and TcWRKY2 showed the highest log_2_ fold change (log_2_FC) values by 12 hours (Fig. 1D and fig. S2B). This temporal pattern suggests that TcMYB3 may be associated with the initial, inductive transcriptional wave of paclitaxel gene expression, while TcMYB4 and WRKY TFs might be involved in a subsequent transcriptional wave, perhaps fine-tuning or sustaining the response. Their MeJA-induced up-regulation was further validated by reverse transcription polymerase chain reaction (RT-PCR; fig. S2C).

### Screening MeJA induced TFs for their ability to regulate key paclitaxel biosynthetic genes

**Fig. 2.**
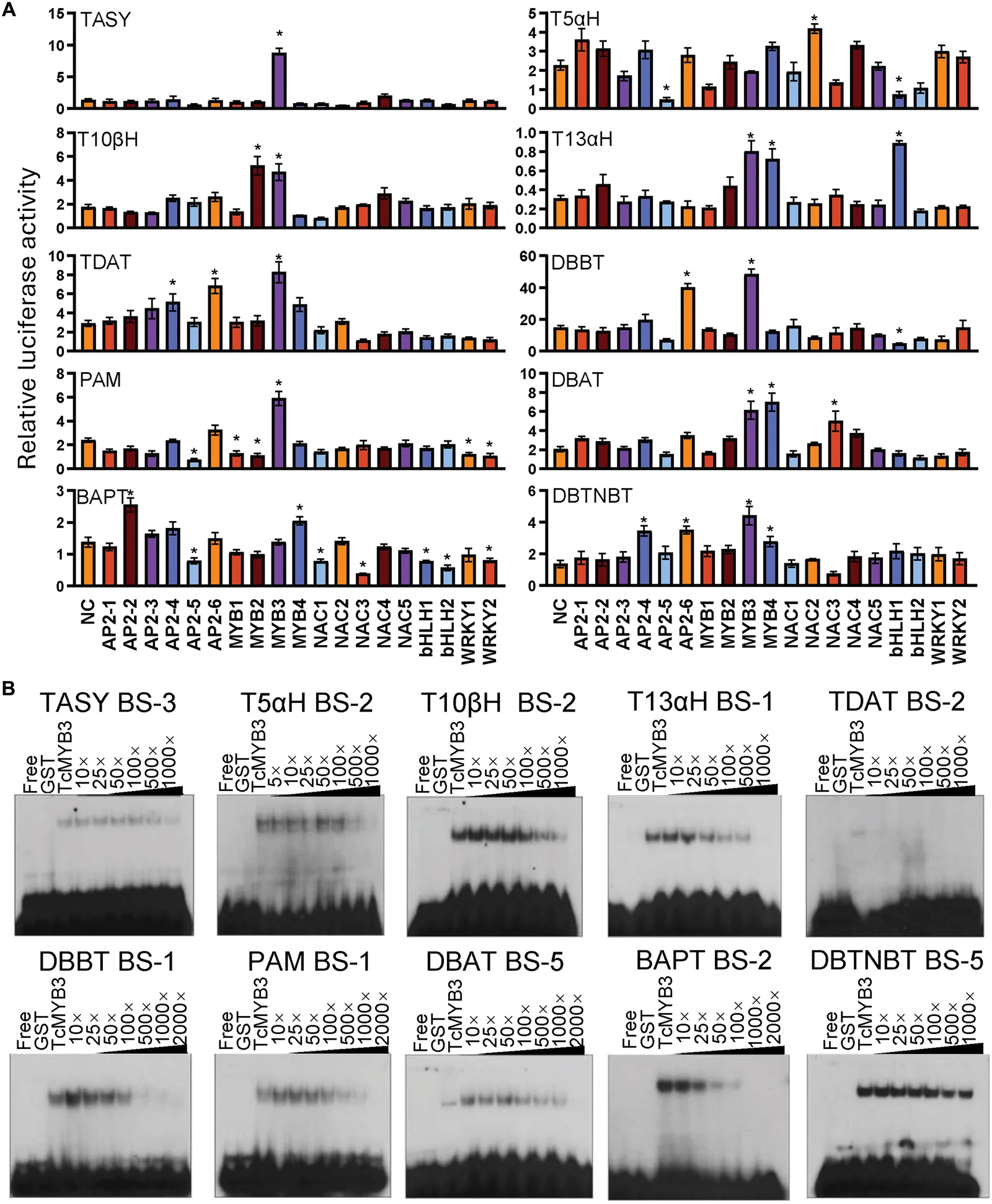
Functional screening of TF candidates by TEA and EMSA. (**A**) *Arabidopsis* transient expression assay (TEA) screen testing for interaction between the 19 candidate TFs and the 10 paclitaxel biosynthetic promoters. Error bars represent standard error; *n* ≥ 4; significance was determined compared to the negative control (NC) in each promoter using a one-way ANOVA following Tukey’s test, *P < 0.05. (**B**) Competitive electrophoretic mobility shift assay (EMSA) for showing the specificity of TcMYB3 to the binding site (BS) in the promoters of 10 paclitaxel biosynthesis genes. Glutathione *S*-transferase (GST) was used as NC. The oligonucleotide probe for each BS is indicated above the respective EMSA panels, and their sequences are detailed in table S4.

To investigate whether these TFs can regulate paclitaxel biosynthesis, we firstly performed in silico analysis using the plant cis-acting regulatory DNA elements tool (*22*) to screen the promoter regions of 10 key paclitaxel biosynthetic genes, TASY, taxadiene-5α-hydrolase (T5αH), T13αH, taxadiene-5α-ol-*O*-acetyl transferase (TDAT), T10βH, DBBT, 10-deacetylbaccatin III-10-*O*-acetyltransferase (DBAT), phenylalanine amminomutase (*23*), Baccatin III:3-amino, 13-phenylpropanoyl transferase (BAPT), and *N*-benzoyltransferase (DBTNBT) (fig. S1E). This analysis identified one or more putative cognate DNA binding sites for each selected TF within the sequence of all available paclitaxel gene promoters, and these motifs were generally overrepresented (fig. S3, A and B). To experimentally determine whether these TFs could interact with paclitaxel gene promoters, we used a high-throughput *Arabidopsis* transient expression assay (TEA) using a dual luciferase reporter system (fig. S3C). Each of the 19 candidate TFs was individually tested for its ability to regulate expression from the 10 paclitaxel gene promoters. This comprehensive screen identified 36 significant interactions, with at least three TFs interacting with more than one promoter, demonstrating both activation and repression (Fig. 2A). Notably, TcAP2-2, TcMYB1, TcNAC1, TcNAC2, bHLH2, and TcWRKY1 showed highly specific interactions, targeting only BAPT, phenylalanine aminomutase (PAM), BAPT, T5αH, BAPT and PAM promoters, respectively. TcAP2-1, TcAP2-3, TcNAC4, and TcNAC5 failed to regulate any of the 10 promoters tested (Fig. 2A). In contrast, nine TFs interacted with multiple promoters, highlighting their broader regulatory potential. We observed that TcMYB2, bHLH1, and TcNAC3 exhibited dual roles as both positive and negative regulators, with their activity being promoter dependent. Significantly, 8 of the top 10 strongest positive interactions were mediated by MYB proteins, with the majority targeting promoters integral to the later steps of the paclitaxel biosynthetic pathway. However, the most potent interaction identified was between TcMYB3 and TASY, which catalyzes the first committed step of the biosynthetic pathway. While no single TF could up-regulate expression of all tested gene promoters required for paclitaxel biosynthesis, TcMYB3 remarkably activated all tested promoters except for T5αH and BAPT, the latter of which is thought to encode a rate-limiting step in paclitaxel biosynthesis (*5*, *6*). Crucially, TcMYB4 demonstrated the ability to up-regulate BAPT. These findings suggest that the combined action of TcMYB3 and TcMYB4 might be sufficient to drive comprehensive expression across key paclitaxel biosynthetic promoters.

### Electrophoretic mobility shift assay confirmed the binding of TcMYB3 and TcMYB4 to paclitaxel biosynthetic genes

To corroborate the direct binding of TcMYB3 and TcMYB4 to the paclitaxel biosynthetic gene promoters, as implied by the TEA, we generated recombinant proteins for these TFs and performed electrophoretic mobility shift assays (EMSAs). This analysis unequivocally established that TcMYB3 strongly binds to the promoter sequences of all 10 key paclitaxel biosynthetic genes (fig. S4). Furthermore, we successfully identified specific binding sites for TcMYB3 within these target promoter sequences (fig. S4). The specificity of these interactions was confirmed by EMSA competitor assays (Fig. 2B). Similarly, TcMYB4 demonstrated robust binding to the promoter sequences of TASY, DBAT, DBBT, DBTNBT, BAPT, and, significantly, T5αH, a cytochrome P450 enzyme that catalyzes a critical early step in the pathway (*16*, *17*). Specific binding sites for TcMYB4 within these target promoter sequences were also identified, and specificity of these interactions was confirmed by EMSA competitor assays (fig. S5). The precise locations of TcMYB3 and TcMYB4 binding sites within these promoters are comprehensively shown in fig. S6A. Detailed analysis of the associated DNA binding sequences revealed the consistent presence of an AC element containing the consensus sequence A(A/C)C (fig. S6B). Collectively, our data demonstrated that TcMYB3 exhibited broad promoter occupancy across the 10 core biosynthetic genes yet notably failed to bind BAPT and T5αH in our TEA assay (Fig. 2B). However, EMSA results revealed that TcMYB4 specifically bound to BAPT and T5αH, effectively complementing TcMYB3’s regulatory scope. These findings establish the TcMYB3-TcMYB4 module as a central positive regulatory unit that coordinately orchestrated the paclitaxel biosynthetic pathway.

### In vivo confirmation that TcMYB3 and TcMYB4 can activate paclitaxel biosynthetic genes

**Fig. 3.**
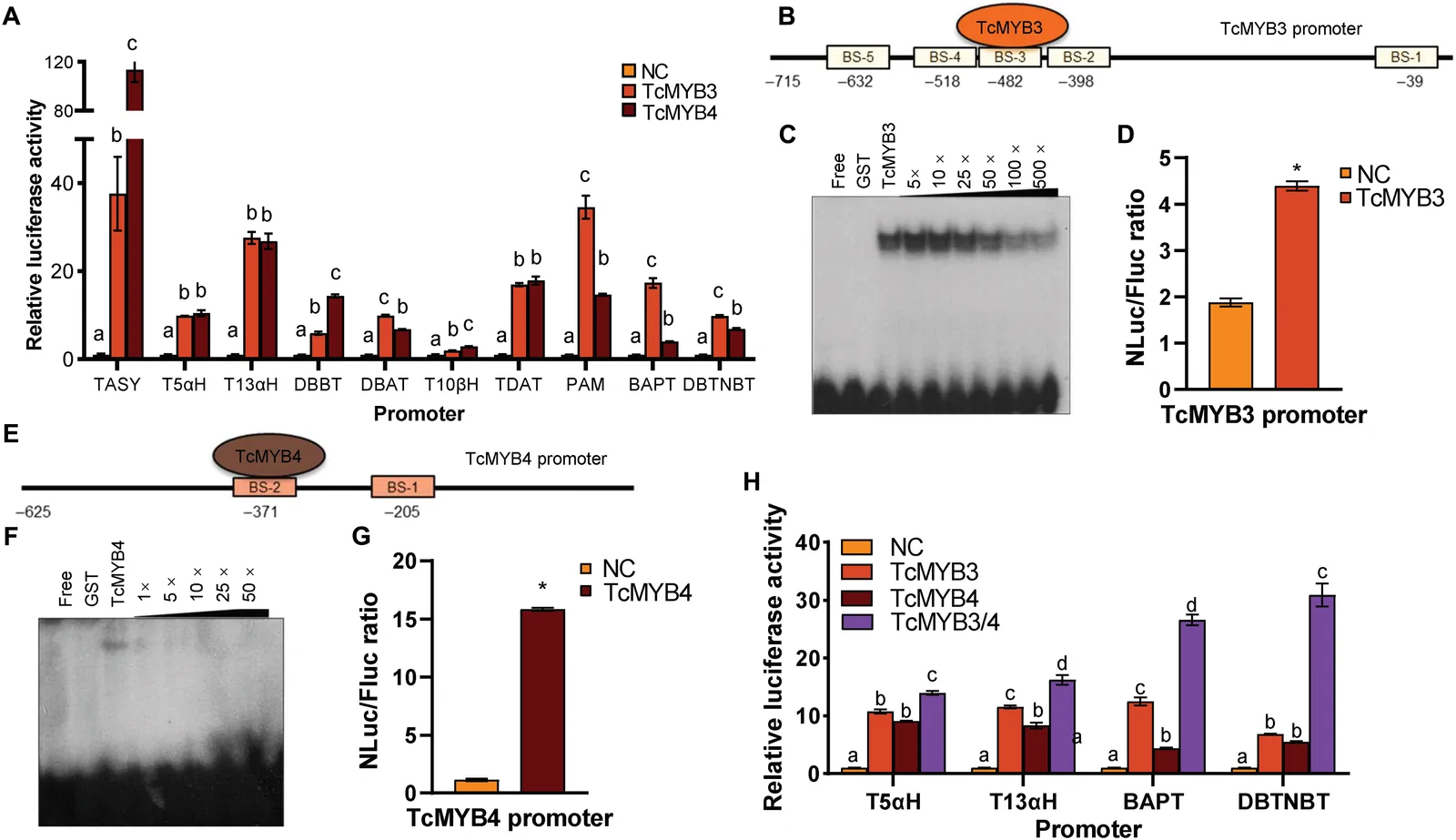
Combinatorial function of TcMYB3 and TcMYB4 in activating paclitaxel biosynthetic enzyme promoters. (**A**) CMC protoplast TEA showing the activation of 10 paclitaxel gene promoters by TcMYB3 driven by *Ubqi10* promoter and TcMYB4 driven by *CaMV 35S* promoter. (**B**) Schematic representation of the TcMYB3 promoter showing the location of the five MYB-BS sites in its promoter. (**C**) Competitive EMSA assay for showing the specificity of TcMYB3 to the BS-3 in its own promoters. Sequences are detailed in table S4. (**D**) CMC protoplast TEA showing the autoactivation of TcMYB3 using the complete TcMYB3 promoter (715 bp). (**E**) Schematic representation of the TcMYB4 promoter showing the location of the MYB-BS site. (**F**) Competitive EMSA assay for showing the specificity of TcMYB4 to the BS-2 in its own promoters. Sequences are detailed in table S4. (**G**) CMC protoplast TEA showing the autoactivation of TcMYB3. (**H**) Combinatorial transactivation of the T5αH, T13αH, BAPT and DBTNBT promoters by TcTMYB3 and TcMYB4. The interactions shown are stated in NLuc/FLuc ratios, normalized against the NC for each promoter. *n* > 3; significance was determined compared to the NC for each transactivator using a Student’s *t* test, *P < 0.05 in [(D) and (G)]. Values are means ± SD, *n* = 3, and different letters indicate significant differences at *P* < 0.05.

To confirm whether TcMYB3 and TcMYB4 can drive the expression of paclitaxel-related genes in *Taxus* cells, we constructed a dual-luciferase reporter vector that consolidated the reporter gene and the internal control into a single plasmid using Mobius Assembly (*24*) (fig. S7, A to C). We evaluated the protoplast transfection in *Taxus* CMCs, determining that 16 μg of DNA achieved the highest transient expression and that the ubiquitin10 promoter (*Ubiq10p*) showed stronger activity compared to *35S* and *MAS* promoters (fig. S7, D and E). This TEA in *T. cuspidata* CMCs demonstrated that TcMYB3 could activate all 10 key paclitaxel biosynthetic gene promoters (Fig. 3A), as suggested by the EMSA (Fig. 2B). However, TcMYB3 failed to activate the promoters of BAPT and T5αH in the heterologous *Arabidopsis* TEA assay (Fig. 2A), highlighting limitations of the heterologous system, possibly due to the absence of essential cofactors or interacting partners native to *Taxus*. Unexpectedly, TcMYB4 also significantly enhanced the activity of all these promoters (Fig. 3A), a result that diverged from the binding observed in EMSA and TEA in *Arabidopsis* (Fig. 2A and fig. S5). Notably, TcMYB4 bound the TASY and T5αH promoters in EMSA but did not activate them in *Arabidopsis* (Fig. 2A and fig. S5), indicating that its DNA binding ability alone is insufficient for transcriptional activation in the heterologous *Arabidopsis* cells. Together, these results demonstrate that the full transcriptional potential of the TcMYB3/4 module is tightly linked to native cellular machinery, underscoring the importance of studying these regulators within their endogenous context.

This intriguing discrepancy prompted us to investigate potential autoregulation and transactivation between TcMYB3 and TcMYB4. TcMYB3 up-regulated expression from its own −398–base pair (bp) promoter and a −525-bp deletion derivative based on the TEA in *Taxus baccata* (*Tb*) CMCs but not from a −258 bp promoter deletion (Fig. 3B and fig. S8, A and B). The −525- to −258-bp TcMYB-3 promoter sequence contains three putative MYB binding sites at −518, −482, and −298 (Fig. 3B). EMSAs confirmed that TcMYB3 specifically bound to the −482 binding site sequence AACACC (Fig. 3, B and C, and fig. S8, A and B). TEA in *T. baccata* CMCs assay confirmed the autoregulation of TcMYB3 using its full-length promoter (Fig. 3D). Autoregulation of the −625-bp TcMYB4 promoter was also observed within −625 bp of its promoter but not with the −278-bp deletion derivative (Fig. 3E and fig. S8C), and EMSA results demonstrated that TcMYB4 could bind its promoter within −371 bp (Fig. 3, E and F, and fig. S8D). TEA in *T. baccata* CMCs assay confirmed the autoregulation of TcMYB4 using its full-length promoter (Fig. 3G). Furthermore, we found that TcMYB4 transactivated the TcMYB3 promoter fragments but not vice versa (fig. S9), suggesting a hierarchical regulatory relationship.

To further evaluate the cooperative function of these MYBs in activating paclitaxel biosynthetic enzyme promoters, we tested combinations of both MYBs in the protoplasts of *T. baccata* CMCs. The coexpression of TcMYB3 and TcMYB4 significantly increased the activation of paclitaxel-related promoters with increased activity being observed for at least four key paclitaxel-related promoters, T5αH, T13αH, BAPT, and DBTNBT (Fig. 3H). These data demonstrate that cotransfection of TcMYB3 and TcMYB4 can increase the activation of T5αH, T13αH, BAPT, and DBTNBT.

### Production of stable transgenic *Taxus* CMCs with identified MYB TFs

**Fig. 4.**
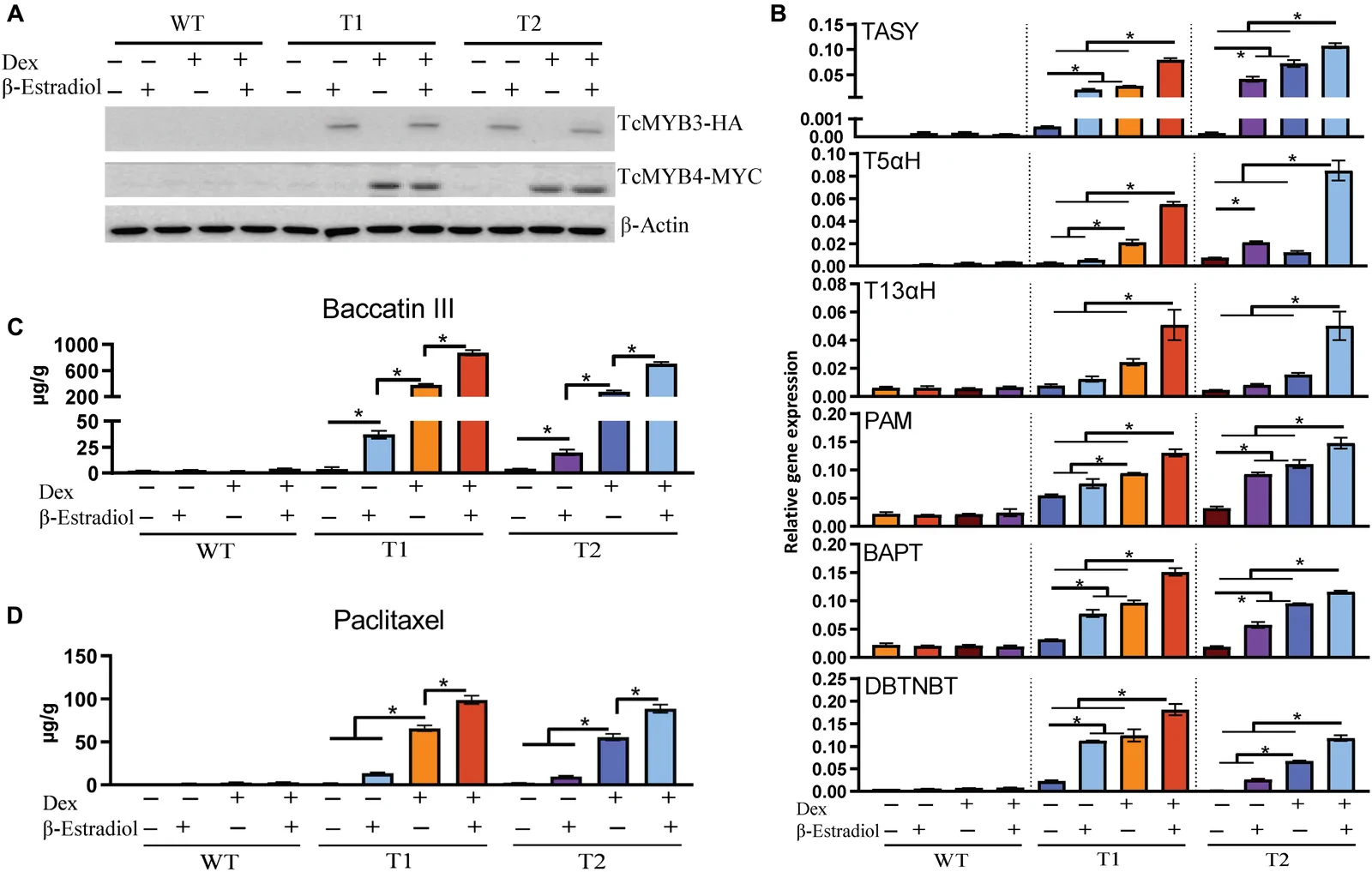
Molecular engineering *Taxus* CMCs cells expressing TcMYB3-TcMYB4 module enhance production of paclitaxel, and its key intermediates baccatin III production. (**A**) Immunoblot of TcMYB3 and TcMYB4 expression in transgenic CMCs lines using hemagglutinin (HA) and MYC antibody, respectively. β-Actin was used as a loading control. The samples were collected after Dex and β-estradiol treatment. WT, wild-type; T1, transgenic line 1; T2, transgenic line 2. (**B**) Transcriptional expression levels of key genes involved in the biosynthetic pathway of paclitaxel in indicated line following a 12-hour treatment with the indicated chemicals. (**C** and **D**) Baccatin III (C) and paclitaxel (D) yields in micrograms per gram of fresh cells following a 7-day treatment with the indicated chemicals. Data are means ± SD; *n* = 3 biological replicates. *P < 0.05.

To determine whether coexpression of TcMYB3 and TcMYB4 in *T. baccata* CMCs was sufficient to drive paclitaxel biosynthesis, we engineered stable transgenic *Taxus* CMC lines. We constructed expression vectors for TcMYB3 and TcMYB4 driven by β-estradiol and dexamethasone (Dex)–inducible promoters, respectively (fig. S10A). Confirmation of TcMYB3 and TcMYB4 expression upon induction with β-estradiol and Dex, respectively, was obtained (Fig. 4A and fig. S10, B to D). Critically, induced expression of either TcMYB3 or TcMYB4 individually activated the expression of paclitaxel biosynthetic enzymes (Fig. 4B and fig. S11A), consistent with our protoplast data (Fig. 3A). Moreover, coexpression of TcMYB3 and TcMYB4 further significantly enhanced the expression of these enzymes compared to individual expression, definitively demonstrating the combinatorial function of TcMYB3 and TcMYB4 (Fig. 4B). Further, coexpression of both TcMYB3 and TcMYB4 in *T. baccata* CMCs resulted in a notable accumulation of the commercially relevant precursor, baccatin III (Fig. 4C) and paclitaxel, FW (98 μg/g), (Fig. 4D), resulting in a 121-fold increase in paclitaxel production and a 364-fold increase in baccatin III production, FW (877 μg/g). Further, the increase in paclitaxel and baccatin III yields following MeJA elicitation in TcMYB3/4-overexpressing lines suggests a combinatorial regulatory effect (fig. S11B), likely driven by the recruitment of endogenous MYB3/4 or by additional unidentified MeJA-responsive activators. Collectively, the combined expression of TcMYB3 and TcMYB4 is able to promote the accumulation of paclitaxel and related taxanes in *Taxus* CMCs, and our data further point to a broader MeJA-responsive transcriptional network that likely functions upstream of, in parallel with, or downstream of the TcMYB3/4 module to drive taxanes biosynthesis.

## DISCUSSION

Commercial supplies of paclitaxel depend on a costly and environmentally unfriendly semisynthesis process that requires the extraction of key intermediates such as baccatin III from *Taxus* species. MeJA is a well-known elicitor of plant natural products and is used commercially to increase paclitaxel production in plant cell culture (*20*, *25*). The identification of putative regulators involved in the up-regulation of paclitaxel production after MeJA elicitation could be exploited in bioengineering efforts to increase paclitaxel yields in plant cell culture.

This study successfully elucidates the transcriptional regulatory mechanism by which MeJA boosts paclitaxel biosynthesis in *Taxus* CMCs. We identified a TcMYB3-TcMYB4 module that functions as an important regulatory switch, concurrently activating 10 essential promoters across the paclitaxel pathway (Fig. 2 and fig. S1E). Stable transgenic *Taxus* CMCs expressing this module exhibited significantly enhanced paclitaxel production (FW, 98 μg/g) (Fig. 4D) to a level equivalent to MeJA elicitation (FW, 102 μg/g) (*20*), demonstrating a critical transition from observing complex cellular responses to designing precise transcriptional control systems. Our previous work established that *Taxus* CMCs exhibit exceptional metabolic robustness during scale-up, with paclitaxel titers increasing from 102 mg/kg in shake flasks to 268 mg/kg in 20 liters of airlift bioreactors upon elicitation for 45 days (*20*). While contemporary industrial company, Phyton Biotech, achieve yields of about 1 to 2 g/kg, these proprietary systems often rely on highly optimized, large-scale bioreactor operations and well-defined elicitation that are finely tuned for their specific cell lines. Various *Taxus* species, such as *Taxus brevifolia*, *T. baccata*, *Taxus cuspidate*, *Taxus chinensis*, and *Taxus media* (a cross of *T. baccata* and *T. cuspidata*), have been explored for paclitaxel and related taxanes production (*25*). Industrial production has most used high-yielding lines derived from *T. chinensis* or *T. media*. In contrast, the present study uses *T. baccata* to generate TcMYB-overexpressing CMCs. It is critical to recognize that productivity varies substantially not only between species but also among individual cell lines, due to distinct selection histories and process optimization. Production from industry typically represent final, optimized titers achieved in large-scale bioreactors over full production cycles (often from 3 to 5 weeks), while the yields reported in our study for transgenic CMCs are from small-scale, nonoptimized shake-flask cultures at a single 7-day time point (Fig. 4D). Therefore, the production levels in our nonoptimized, research-stage CMC line are not directly comparable to those of an industrial cell factory. The key finding is the significant fold increase over controls achieved through genetic engineering, which establishes a foundation for future process optimization. The translational value of our discovery lies in providing a genetic tool (TcMYB3/4) that can be applied to enhance yields in any chosen, high-performing production line, irrespective of its specific taxonomic origin or optimization background.

In contrast, the CMC platform provides a highly sustainable and nearly inexhaustible biomass source. The characterization of the R2R3-MYB TFs in this study provides a critical molecular toolkit to bridge the current yield gap. By inducible overexpressing these positive regulators, we can genetically stabilize a high-productivity phenotype, securing the long-term viability of this platform while maintaining the biological longevity of the cell lines. Furthermore, while we used a β-estradiol–inducible system for this proof of concept, transitioning to Dex or ethanol-inducible systems would be more appropriate for clinical-grade industrial applications to avoid potential regulatory complexities associated with estrogen receptor–positive medical contexts (*26*).

Metabolic engineering in heterologous hosts has successfully demonstrated the de novo biosynthesis of paclitaxel and its key intermediate, baccatin III, the industrial precursor to paclitaxel biosynthesis (*12*). Significantly, the key enzymes required for baccatin III and paclitaxel biosynthesis, including several “missing” genes have been identified, allowing reconstitution of the de novo baccatin III biosynthesis pathway (*5*, *15*, *27*). For example, the minimal gene set required for paclitaxel biosynthesis was constructed in *N. benthamiana*, enabling baccatin III levels to reach 154.84 ng/g, close to the amount produced in *Taxus* needles (225.32 ng/g) (*18*). Moreover, a 17-gene biosynthesis of baccatin III in *Nicotiana* was constructed and resulted in baccatin III production at levels comparable to its natural abundance in *Taxus* needles (*5*). Although the paclitaxel biosynthetic pathway has been successfully elucidated and engineered in heterologous hosts, translational application is limited by an inability to impose native regulatory control over metabolic flux and the characteristically inefficient functional expression of cytochrome P450 enzymes (*12*, *17*, *28*, *29*). This limitation is further exemplified by our finding that TcMYB3, despite activating all key promoters in its native context, failed to activate the promoters of BAPT and T5αH in the heterologous *Arabidopsis* TAE assay (Fig. 2A), underscoring the constraints of non-native systems, likely due to the absence of essential *Taxus*-specific cofactors or interacting partners. In contrast, the native *Taxus* cell culture remains a promising alternative, offering a more sustainable production method, and metabolic engineering in host cells offers a scalable and environmentally conscious alternative (*19*). For example, culture optimization in combination with elicitation can promote production of paclitaxel, giving a content of 43.43 mg/liter (*30*), and overexpression of BAPT and DBTNBT genes in *T. baccata* cells leads to production of taxane at 53.83 mg/liter (*31*). Here, we identified the TF network integral to paclitaxel biosynthesis, and bioengineering *Taxus* CMCs cells expressing this selected TFs could enhance production of paclitaxel, reaching 98 μg/g FW, about 121 times higher than the levels seen in control cells (Fig. 4D), and the key intermediate baccatin III, 877 μg/g FW, about 364 times higher compared to the control (Fig. 4C), and much higher than previously reported values for heterologous hosts and other cell culture (*32*).

TF engineering represents a highly promising strategy for enhancing metabolite production, as demonstrated by its successful application in directing the flux of fungal polyketide biosynthesis for lovastatin production and in reconstructing complete anthocyanin pathways to create anthocyanin-enriched tomato fruit (*33*–*35*). Prior work in paclitaxel biosynthesis engineering has often relied on overexpressing single TF, such as MYB, bHLH, or NAC factors, including those recently identified, such as *T. chinensis* MYB29a, *Taxus media* TmMYB3, or TmMYB39-TmbHLH13 (*32*, *36*, *37*). While these efforts have yielded important insights into paclitaxel regulatory points, they have consistently struggled to achieve the sustained, multienzyme coordination necessary for high-level metabolite production. This limitation stems from relying on piecemeal approaches that fail to orchestrate the simultaneous activation of the entire biosynthetic pathway in heterologous hosts (*13*). However, our work demonstrates the successful de novo identification and construction of a biosynthesis-optimized TF network. We initiated an unbiased screen of candidate TFs, and this systems-level approach allowed us to move beyond targeted, gene-by-gene investigations and instead identify a minimal subset of TFs capable of collectively enhancing the entire pathway. The in vivo validation of this TF network in host cells, resulting in a substantial and measurable enhancement of paclitaxel biosynthesis (Figs. 2 and 3), underscores the practical utility and transformative potential of our findings. The key conceptual breakthrough of our study is moving beyond the characterization of known TFs to the successful de novo identification and construction of a biosynthesis-optimized TF network within the host cell. This demonstration of concerted regulatory action offers a more potent and coordinated strategy than traditional methods that target individual enzymes, representing a paradigm shift toward systems-level control of plant metabolism.

Although our work could substantially enhance the production of paclitaxel and its commercial intermediate baccatin III, several strategic avenues promise to further amplify the production platform based on our work. While the coexpression of TcMYB3 and TcMYB4 is sufficient to strongly induce the biosynthesis of paclitaxel and related taxanes in *Taxus* CMCs, we do not propose that this module represents the sole or master regulator of the MeJA-induced paclitaxel biosynthesis. Instead, our data indicate the existence of a broader, combinatorial transcriptional network. The synergistic increase in paclitaxel and baccatin III accumulation following MeJA elicitation in TcMYB3/4-overexpressing lines (fig. S11B) suggests that additional, unidentified MeJA-responsive TFs, which may function upstream of, in parallel with, or downstream of the TcMYB3/4 module, cooperate to achieve full pathway activation. Among the candidate coregulators, the functional homolog of the jasmonate signaling master regulator, bHLH TF AtMYC2 (*38*), represents a particularly compelling target for future mechanistic investigation. Building on our work, several targeted strategies emerge to boost paclitaxel. First, a deeper molecular understanding of the TcMYB3-TcMYB4 network, including the identification of their protein interactors and the elucidation of how their binding specificities are integrated during MeJA elicitation, may reveal endogenous coregulators to recruit or suppress for further bioengineering. Second, identifying the endogenous repressors that attenuate the MeJA response presents a target for CRISPR-Cas9–mediated knockout to amplify pathway induction. Additional study could focus on knockout of competing side pathways, such as the phenylpropanoid pathway, which would channel metabolic flux more exclusively toward paclitaxel (*39*, *40*). Last, coupling this genetic blueprint with optimized culture conditions will be essential for scalable bioprocessing. Ultimately, this work establishes rational TF network engineering as a foundational strategy, heralding a previously unexplored era for the sustainable production of complex plant natural products.

In summary, our study demonstrates the potential applications of transcriptional network engineering for improving the production of valuable plant secondary metabolites. We anticipate that a similar systematic identification and characterization of other TF networks will pave the way for generating robust, high-yield production systems for a wide range of natural products.

## MATERIALS AND METHODS

### Transcriptomic profiling

Differential expression analysis was performed using DESeq2 on the dataset with biological replicates (*41*). Genes with an adjusted *P* value (*P*_adj_) < 0.05 and |log_2_FC| > 1 were considered differentially expressed in MeJA-treated samples. For each ontology, enrichment was computed against the background of all annotated contigs, and significance was assessed using Fisher’s exact test, followed by Benjamini-Hochberg false discovery rate (FDR) correction. Venn diagrams were generated using the VennDiagram package to display overlap of DEGs across time points. DEG counts per time point were visualized by bar plots. Heatmaps of the union of DEGs were generated using ComplexHeatmap, with *z*-score–scaled log_2_FCs, clustering by Euclidean distance. Time-course clustering of DEGs was performed using *k*-means (*k* = 6). Cluster trends were visualized with smoothed line plots of *z*-score expression trajectories. Overrepresentation analysis was conducted separately for Biological Process (BP), Molecular Function (MF), and Cellular Component (CC) using Fisher’s exact test with Benjamini-Hochberg FDR correction. The top 10 significant GO terms were plotted as dot plots.

### Genomic DNA extraction

Approximately 100 mg of frozen tissue was ground in liquid nitrogen, suspended in extraction buffer, and incubated at 65°C for 30 min. An equal volume of chloroform:isoamyl alcohol (24:1, v/v) was added, mixed, and centrifuged at 10,000*g* for 10 min at 4°C. The aqueous phase was reextracted, and DNA was precipitated with 1.5 volumes of isopropanol at 20°C for 1 hour. DNA was pelleted (17,000*g* for 30 min at 4°C), washed with 70% ethanol, air dried, and resuspended in 50 μl of Tris-EDTA (TE) buffer at 65°C for 5 min.

### Rapid amplification of cDNA ends

Rapid amplification of cDNA ends (RACE) was used to obtain full-length sequences of genes with incomplete coding regions. Five-prime RACE was performed for *TcAP2-3*, *TcNAC3*, and *TcbHLH2*, and three-prime RACE was performed for *TcMYB4*. Amplified cDNA fragments were cloned and sequenced using the 5′/3′ RACE Kit, Second Generation (Roche), following the manufacturer’s instructions. Primer sequences are listed in table S1.

### Gene expression analysis

*T. cuspidata* CMCs were collected in the indicated time in the figure legend, and total RNA was extracted using a modified cetyltrimethylammonium bromide (CTAB) method (*42*). Total RNA was deoxyribonuclease treated and reverse transcribed using oligo(dT) primers and the First-Strand cDNA Synthesis Kit (Invitrogen). Quantitative RT-PCR was performed with gene-specific primers (table S2) and others from (*43*) on a LightCycler 480 (Roche). Reactions were run in triplicate across three biological replicates. Relative expression was normalized to *Taxus* actin, and significance determined using one-way analysis of variance (ANOVA) following Tukey’s test (*P* < 0.05).

### Plasmid construction for transient expression in *Arabidopsis thaliana*

The *Renilla* luciferase sequence from pRL-TK (Promega) was inserted into p2GW7.0 to generate an internal control plasmid. TF coding sequences were amplified from cDNA, and promoter fragments were amplified from genomic DNA using Phusion High-Fidelity DNA Polymerase (New England Biolabs). TFs were cloned into p2GW7.0 to produce effector constructs, and promoters were cloned upstream of the firefly luciferase gene in pGWlucB to produce reporter constructs. Primer sequences are provided in table S3.

### *A. thaliana* protoplast transient assay

Ten *Taxus* promoters and 19 TFs were cloned using the Gateway system (table S3). Protoplast transfection followed an optimized protocol (*44*). A mixture of 250 μl of protoplasts (5 × 10^5^ cells/ml) and 14 μl DNA (1 μg/μl; effector:reporter:control = 4:5:5) was incubated with polyethylene glycol (PEG)–Ca^2+^ solution [40% PEG 4000, 0.4 M mannitol, and 0.1 M Ca(NO_3_)_2_ (pH 8 to 9)] for 5 min at room temperature. Cells were washed, incubated in modified W5 for 20 hours at 25°C under light, frozen in liquid nitrogen, and stored at −80°C. Dual-luciferase assays were conducted using the Promega system. Each experiment was repeated three times. Positive interactions were defined by significant induction relative to the empty-vector control (*P* < 0.05).

### Expression and purification of recombinant TcMYB3 and TcMYB4

The coding sequences of TcMYB3 and TcMYB4 were cloned into the glutathione *S*-transferase (GST)–tagged vector pDEST24 using the primers in table S3 and expressed in *E. coli* BL21 (DE3). Protein expression was induced at optical density at 600 nm (OD_600_) = 0.6 to 0.7 with 0.1 mM isopropyl-β-d-thiogalactopyranoside at 28°C for 3 hours. Cells were lysed in buffer [25 mM tris-HCl (pH 7.5), 100 mM NaCl, 0.1% Triton X-100, lysozyme (1 mg/ml), 1 mM dithiothreitol (DTT), and protease inhibitor], and GST-fusion proteins were purified using Glutathione Sepharose 4B (GE Healthcare); the GST was used as a control.

### Electrophoretic mobility shift assay

EMSA was performed using 20-bp double-stranded oligonucleotides containing cis-elements from paclitaxel gene promoters (table S4). Probes were end-labeled with [γ-^32P^]ATP (adenosine triphosphate labeled with phosphorus-32 on the terminal phosphate) using T4 polynucleotide kinase (Fermentas, UK). Binding reactions contained 0.5 μg of recombinant protein in buffer [20 mM Hepes (pH 7.9), 50 mM KCl, 5 mM MgCl_2_, 1 mM DTT, 5% glycerol, poly(deoxyinosinic-deoxycytidylic acid) (0.1 μg/μl), and bovine serum albumin (BSA; 50 μg/μl)]. After incubation (20 min at 4°C; 20 min at room temperature), reactions were loaded on 6% nondenaturing polyacrylamide gels in 1× tris-borate EDTA and run at 120 V for 80 min. Gels were dried and exposed overnight at −80°C to x-ray film.

### Thermal Asymmetric Interlaced-PCR

Promoters of TcMYB3 and TcMYB4 were amplified by thermal asymmetric interlaced PCR as described by Thanh *et al.* (*45*). Briefly, 50 ng of *T. cuspidata* CMC genomic DNA was amplified in three consecutive reactions using nested gene-specific primers (RSP1, RSP2, and RSP3) and arbitrary degenerate primers (table S5). Products were cloned into pGEM-T Easy (Promega) and sequenced. The identified promoter sequences were reamplified and cloned into the Mobius Assembly universal acceptor vector.

### Mobius assembly cloning

Mobius Assembly (*24*) was used to generate constructs for transient expression in *Taxus* CMC protoplasts and for stable transformation. Promoters were fused to nanoluc luciferase (NLuc), while TcMYB3 or TcMYB4 were driven by the *P_LexA_* promoter and *P_pop6_* promoter, respectively. Three level 1 transcriptional units were assembled into level 2 vectors containing the transactivator (TcMYB3 or TcMYB4), promoter-NLuc fusion, and firefly luciferase (FLuc) reporter (fig. S7). The construct used for stable transformation is shown in fig. S10A.

### *Taxus* CMC protoplast isolation and transfection

One milliliter of packed *T. cuspidata* CMCs was digested in 10 ml enzyme solution [1% cellulase Onozuka R-10, 0.25% pectolyase Y-23, 0.05% BSA, and 0.01% MES in maceration-glucose-glycine (MGG) buffer] for 3 hours in darkness. The digest was filtered through a 40-μm nylon mesh, and protoplasts were pelleted (100 g for 3 min) and washed twice with chilled MGG. Cells were resuspended in 1 ml of MGG on ice for 1 hour and adjusted to 5 × 10^5^ cells/ml in MMM solution (0.5 M mannitol, 15 mM MgCl_2_, and 0.1% MES). Cell viability was assessed with Evans blue and fluorescein diacetate. Transfection was performed with 8 μg of DNA, 250 μl of protoplast suspension, and 250 μl of PEG solution [40% PEG 4000, 0.4 M mannitol, and 0.1 M Ca(NO_3_)_2_] for 1 min, followed by dilution with MGG and incubation in darkness for 1 hour. Cells were washed and resuspended in 500 μl of MGG for overnight incubation before luciferase analysis.

### Dual-luciferase assay

Luciferase activity was quantified using the Nano-Glo Dual-Luciferase Reporter Assay System (Promega). Eighty microliters of passive lysis buffer were added to each sample, followed by 80 μl of ONE-Glo EX reagent for firefly luciferase and 80 μl of NanoDLR Stop & Glo reagent for NanoLuc measurement. Luminescence was measured on a BMG Labtech microplate reader (gain, 3600; integration, 1 s). Results were expressed as NLuc/FLuc ratios normalized to controls. Experiments were repeated twice with three replicates per treatment. Statistical significance was determined using Student’s *t* test (*P* < 0.05).

### Stable transformation of *Taxus* CMCs and Western blotting

*Agrobacterium tumefaciens* strain AGL1 was cultured in yeast extract peptone (YEP) medium with rifampicin (25 mg/liter) and kanamycin (50 mg/liter) at 28°C for 2 to 3 days. One milliliter of aliquot was transferred to 20 ml of YEP containing antibiotics and 200 μM acetosyringone and grown to OD_600_ = 0.8. Cells were pelleted (3270*g* for 5 min) and resuspended in infiltration buffer [10 mM MES (pH 5.6), 10 mM MgCl_2_, and 250 μM acetosyringone]. After 1 to 4 hours of incubation at 22°C, 0.02% Silwet-77 was added, and 50 ml of bacterial suspension was mixed with 50 ml of *T. cuspidata* CMCs. Cultures were incubated at 25°C in darkness (110 rpm for 30 min), washed twice with H_2_O, and cocultured in TB medium for 3 days. Cells were washed repeatedly and plated on TB agar containing 400 μM timentin for 7 days, followed by stepwise selection on TB agar with decreasing timentin (300 to 100 μM) and increasing hygromycin (10 to 25 μg/liter) to isolate stable transformants. TcMYB3 or TcMYB4 proteins were detected using anti-Myc (1:2000; Amsbio) or anti-hemagglutinin (HA; 1:2000; Abcam) primary antibodies and IRDye 800CW–conjugated goat anti-mouse or goat anti-rabbit secondary antibodies (1:5000; Cell Signaling Technology).

### Extraction and quantification of taxanes

The CMC cells were treated with concentration of 50 μM β-estradiol, 50 μM Dex, or 100 μM MeJA for indicated times in the figure. Approximately 50 mg of frozen cells were extracted with 1 ml methanol:dichloromethane (1:1, v/v) by sonication for 2 hours. The supernatant was evaporated under vacuum, and the residue was reextracted twice with dichloromethane:H_2_O (2:1, v/v). The combined organic fractions were dried and dissolved in methanol for high-performance liquid chromatography (HPLC)–mass spectrometry (MS) analysis.

Paclitaxel and baccatin III were quantified using a 6560 Ion Mobility Quadrupole Time-of-Flight MS coupled to an Infinity II 1290 UPLC (Agilent) equipped with an ACE C18-PFP column (100 × 2.1 mm, 1.8 μm). A water (A)–methanol (B) gradient containing 0.1% formic acid was used (10 to 100% B over 7 min, held for 4 min, 0.3 ml/min, 30°C). Data were acquired in positive ion mode (mass/charge ratio, 400 to 1000; 1 frame/s). [M + Na]^+^ ions monitored: baccatin III, 609.2306; paclitaxel, 876.3202. Standard curves were prepared from reference compounds (Cambridge Bioscience).

### Statistical analysis

Data were presented as means ± SD. One-way ANOVA followed by Tukey’s test or *t* test were used for the statistical analysis unless other stated. *P* < 0.05 was considered as significant.
