# Supplementary material for: A minimal transcription factor network is sufficient to drive paclitaxel biosynthesis
Source: Sci Adv. 2026 Jun 5;12(23):eaee6211. doi: 10.1126/sciadv.aee6211 (PMC13240184; doi:10.1126/sciadv.aee6211)
Supplement: Supplementary file 1 — Figs. S1 to S11 Tables S1 to S5 [file sciadv.aee6211_sm.pdf]

Science Advances
AAAS

Supplementary Materials for

A minimal transcription factor network is sufficient to drive paclitaxel biosynthesis

Qiaona Pan *et al.*

Corresponding author: Beimi Cui, beimi.cui@gmail.com

Sci. Adv. **12**, eaee6211 (2026)
DOI: 10.1126/sciadv.aee6211

This PDF file includes:

Figs. S1 to S11
Tables S1 to S5

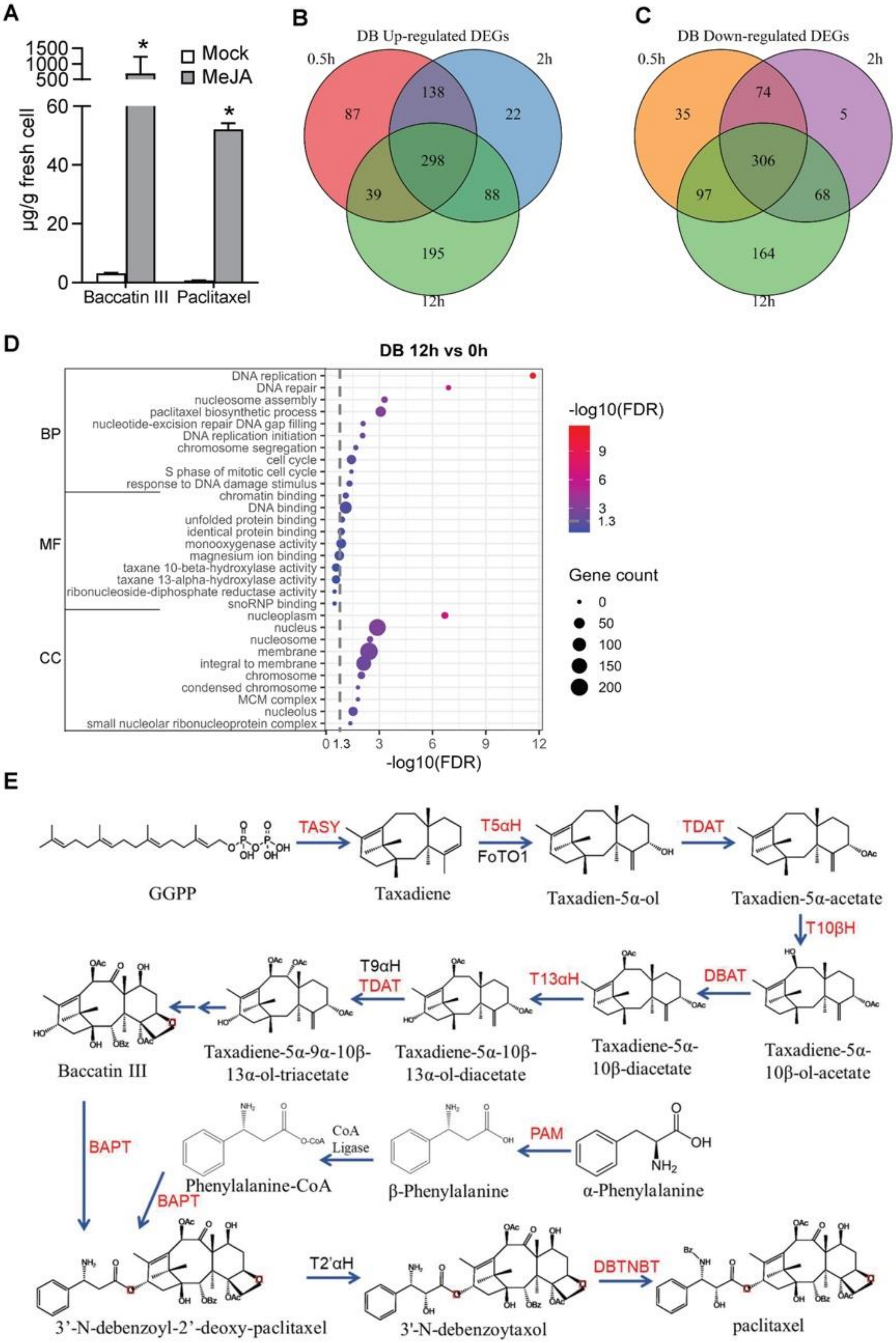

A
B
C
D
E
Mock
MeJA
μg/g fresh cell
Baccatin III
Paclitaxel
DB Up-regulated DEGs
DB Down-regulated DEGs
0.5h
2h
12h
DB 12h vs 0h
BP
MF
CC
-log10(FDR)
Gene count
DNA replication
DNA repair
nucleosome assembly
paclitaxel biosynthetic process
nucleotide-excision repair DNA gap filling
DNA replication initiation
chromosome segregation
cell cycle
S phase of mitotic cell cycle
response to DNA damage stimulus
chromatin binding
DNA binding
unfolded protein binding
identical protein binding
monooxygenase activity
magnesium ion binding
taxane 10-beta-hydroxylase activity
taxane 13-alpha-hydroxylase activity
ribonucleoside-diphosphate reductase activity
snoRNP binding
nucleoplasm
nucleus
nucleosome
membrane
integral to membrane
chromosome
condensed chromosome
MCM complex
nucleolus
small nucleolar ribonucleoprotein complex
GGPP
TASY
Taxadiene
T5αH
FoTO1
Taxadien-5α-ol
TDAT
Taxadien-5α-acetate
T10βH
DBAT
Taxadiene-5α-10β-ol-acetate
T13αH
Taxadiene-5α-10β-diacetate
T9αH
TDAT
Taxadiene-5α-10β-13α-ol-diacetate
Taxadiene-5α-9α-10β-13α-ol-triacetate
Baccatin III
BAPT
CoA Ligase
Phenylalanine-CoA
PAM
β-Phenylalanine
α-Phenylalanine
BAPT
3'-N-debenzoyl-2'-deoxy-paclitaxel
T2'αH
3'-N-debenzoytaxol
DBTNBT
paclitaxel

Fig. S1. Transcriptomic changes in CMC cells after MeJA treatment.
(A) Baccatin III and Paclitaxel yields in μg/g fresh cells following a 7-days treatment with DMSO (Mock) or 100 μM MeJA. Data are mean ± s.d., n = 3 biological replicates. Significance was determined using *t*-test, * p value < 0.05.
(B-C) Venn diagrams of differentially expressed genes (DEGs) across time points (0.5 h, 2 h, 12 h) in *Taxus* cambial meristematic cells (CMCs) upon MeJA treatment.
(D) Dot plots showing the top 10 enriched Gene Ontology (GO) categories for Biological Process (BP), Molecular Function (MF), and Cellular Component (CC) terms among DEGs at 12 h after MeJA treatment compared to 0 h. Circle size represents the number of DEGs annotated to the term, and color intensity indicates statistical significance ($-\log_{10}$ FDR). **(E)** Biosynthetic pathway of paclitaxel from GGPP. This diagram illustrates the sequential enzymatic steps, including core taxane skeleton formation, multiple hydroxylations, and side-chain attachment, leading to the production of the anti-cancer drug paclitaxel. Enzyme genes studied in this work are highlighted in red and this figure was adapted from references (*3-5, 15*).

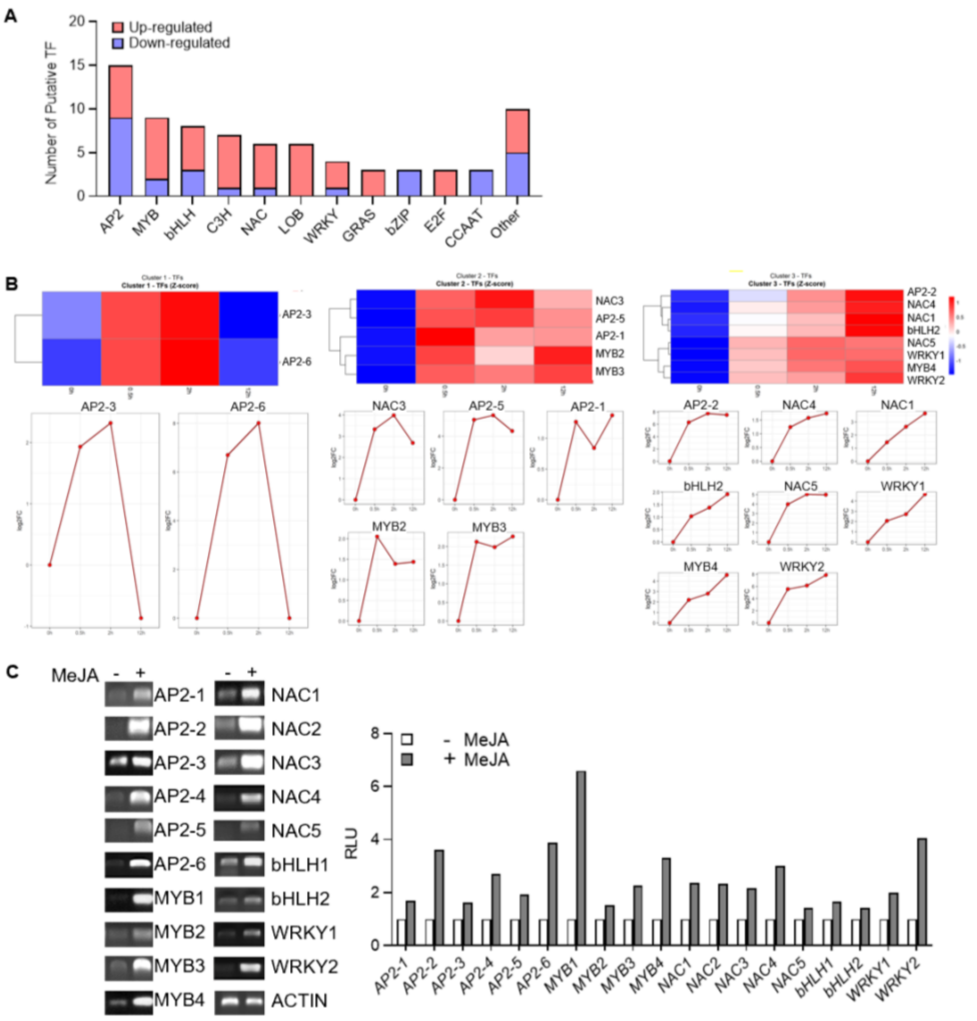

Fig. S2. Characterization of transcription factors (TFs) from transcriptomic analysis.

(A) Distribution of TF families identified from differentially expressed genes (DEGs) in response to MeJA treatment. **(B)** Clustering of TF candidate expression profiles across MeJA treatment time points (0.5 h, 2 h, 12 h). **(C)** RT-PCR validation of selected TFs showing induced expression by MeJA treatment for 0.5 h. Transcripts of selected TFs in CMC cells were determined by semi-quantitative RT-PCR in response to MeJA (+) or DMSO (-) (left panel) and quantitative analysis of RT-PCR bands shown in the left panel (right panel). The relative intensity units (RLU) of the mock-treated control (-) was set to 1.0, and the intensity of the MeJA-treated band (+) was normalized to their respective controls. ACTIN was used as an internal control.

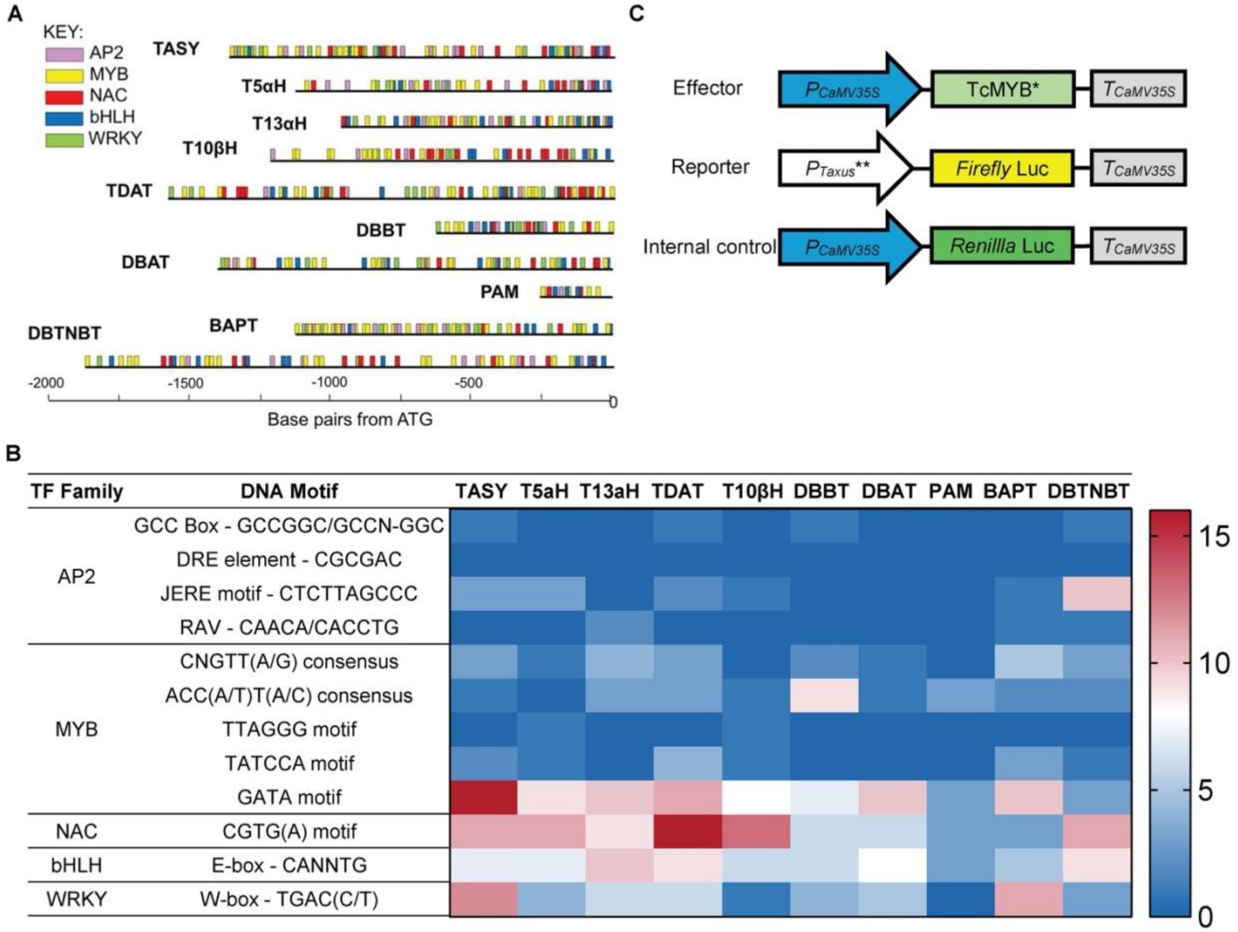

Fig. S3. Summary of the binding site (BS) analysis of promoter by candidate TF and transient expression assay design.

(A) A schematic representation of the predicted binding sites (BSs) for the AP2, MYB, NAC, bHLH and WRKY transcription factor (TF) families in 10 paclitaxel biosynthetic promoters. BSs are indicated by boxes, colour coded based on TF family, AP2 purple, MYB yellow, NAC red, bHLH blue and WRKY green. The image shows the length of each promoter and location of BSs relative to the start codon (ATG). **(B)** Summary of the BS analysis of 10 paclitaxel biosynthetic promoters. BSs were identified with the aid of the PLACE (Plant cis-acting regulatory DNA elements) tool. The name of the BS and the consensus motif are stated for each TF family. The number of times a binding site occurs in a promoter in the 5’ and 3’ direction is stated. The heatmap displays the frequency of these motifs (0–15, as indicated by the blue-to-red scale) within the 2,000 bp upstream regions of 10 core biosynthetic genes, accounting for occurrences on both the sense and antisense strands. The paclitaxel promoters are rich in cognate BSs of the 19 candidate TFs. **(C)** Schematic structure of the DNA constructs integral to the transient expression assay (TEA). The effector construct has the transcription factor of interest (TF) under the control of *CaMV35S* promoter. Reporter plasmid has the promoter under investigation driving the reporter gene Firefly luciferase. The *CaMV35S* promoter drives expression of Renilla luciferase, which functions as an internal control.

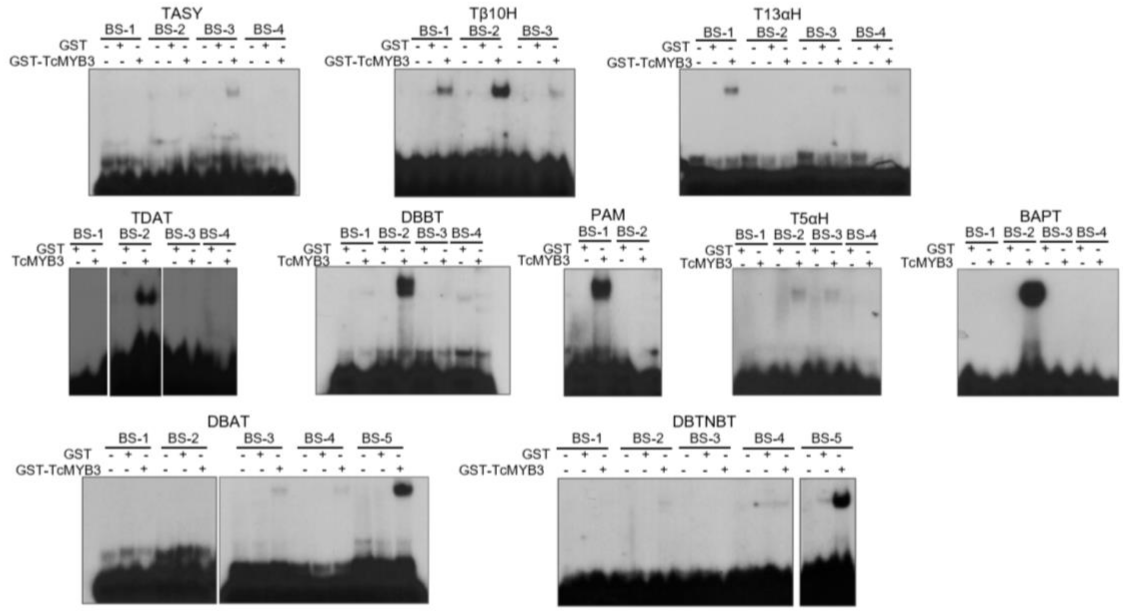

Fig. S4. TcMYB3 directly binds promoter elements of paclitaxel biosynthetic genes. Electrophoretic mobility shift assay (EMSA) using the recombinant protein TcMYB3 to screen the *cis*-element from the 10 indicated paclitaxel biosynthetic genes. Glutathione S-transferase (GST) was used as negative control. The oligonucleotide probe sequences are detailed in Table S4.

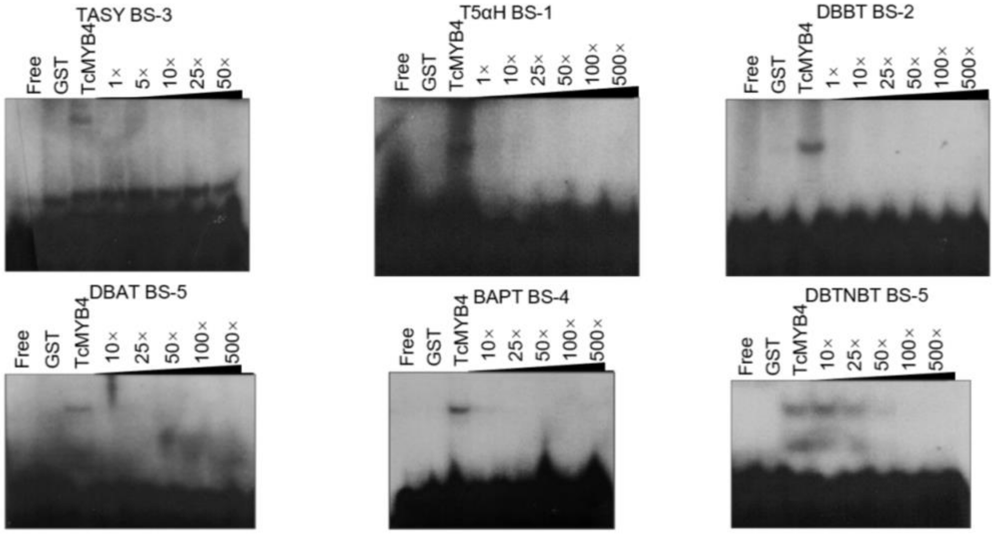

Fig. S5. Competitive EMSA assay for showing the specificity of TcMYB4 to the BS in the promoters of 6 indicated paclitaxel biosynthesis genes. Binding specificity was evaluated by competition assays using increasing concentrations of unlabeled competitor probes. GST was used as negative control. The oligonucleotide probe for each binding site (BS) are indicated above the respective EMSA panels, and their sequences are detailed in Table S4.

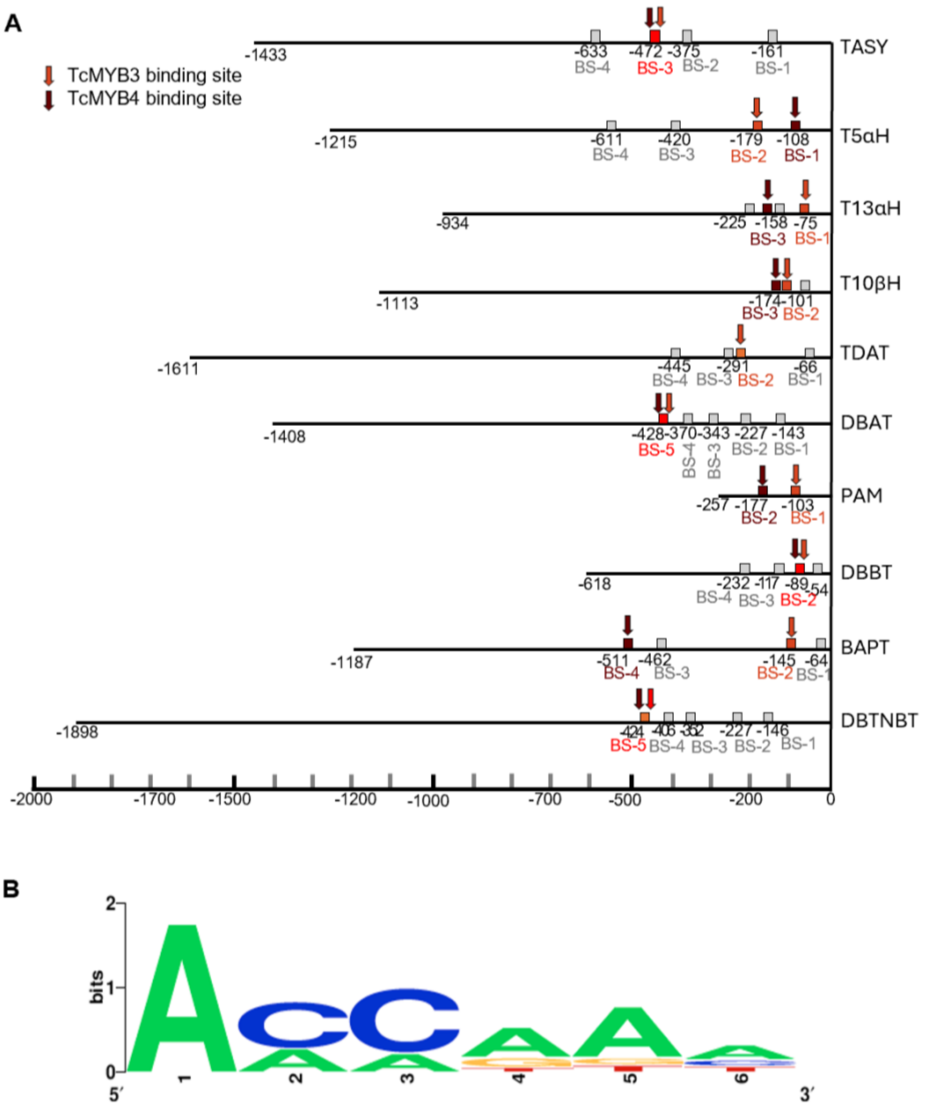

Fig. S6. DNA binding sequences containing the *cis*-elements and regulatory motif for the binding of the TcMYB3 and TcMYB4 protein in all the 10 promoters.

(A) Summary of the location of the binding of the TcMYB3 and TcMYB4 proteins to the *cis*-elements containing the AC motif. Boxes above the promoters indicate the location of the *cis*-element analyzed with EMSA. Orange arrow means TcMYB3 bind to that specific motif as suggested by EMSA. Brown arrows mean TcMYB4 binding site. Binding site (BS) are indicated below the respective promoter were analyzed by EMSA in Fig. 2B, Fig. S4-5 and their sequences are detailed in Table S4. **(B)** Consensus motif recognized by binding specificity of TcMYB3 and TcMYB4 of the 10 DNA sequences from promoter of the paclitaxel biosynthetic gene.

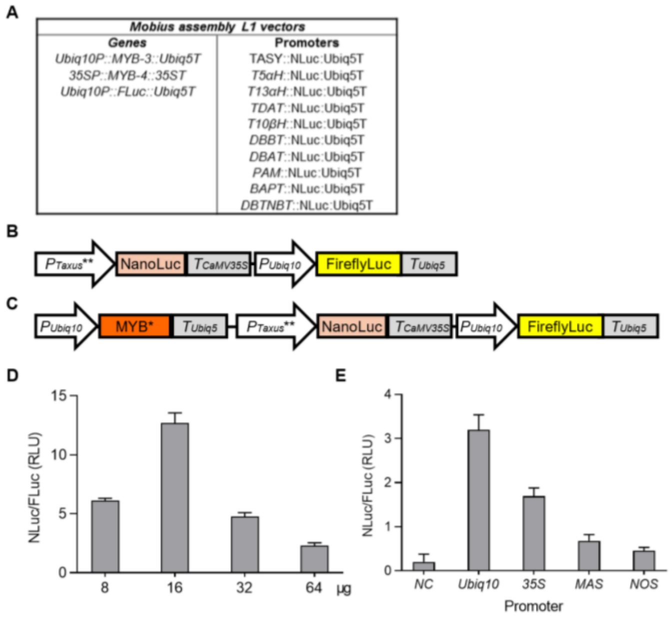

Fig. S7. Optimization of the transfection conditions for a CMC protoplast transient assay.
(A) List of the modular components in the transcriptional units (TUs) for the L1 cloning vectors used for mobius assembly. **(B)** Positive control vector co-expressing NanoLuo (Nluc) and Firefly Luciferase (FLuc) to validate the transfection efficiency. **(C)** Constructs designed for *Taxus* CMC TEA. *MYB**, represents either *TcMYB3* or *TcMYB4*. P_{Taxus}** denotes any of the 10 *Taxus* promoters shown in A. **(D)** Evaluation of positive control vector concentration for transfection efficiency in CMC protoplasts. **(E)** Evaluation of the expression of constitutive promoters, *Ubiq10*, *CaMV35S* (*35S*), *MAS* and *NOS* in CMC protoplasts. Reporter activity is normalized to NanoLuc/Firefly Luciferase (Nluc/FLuc) luminescence ratio. NC = negative control (no plasmid).

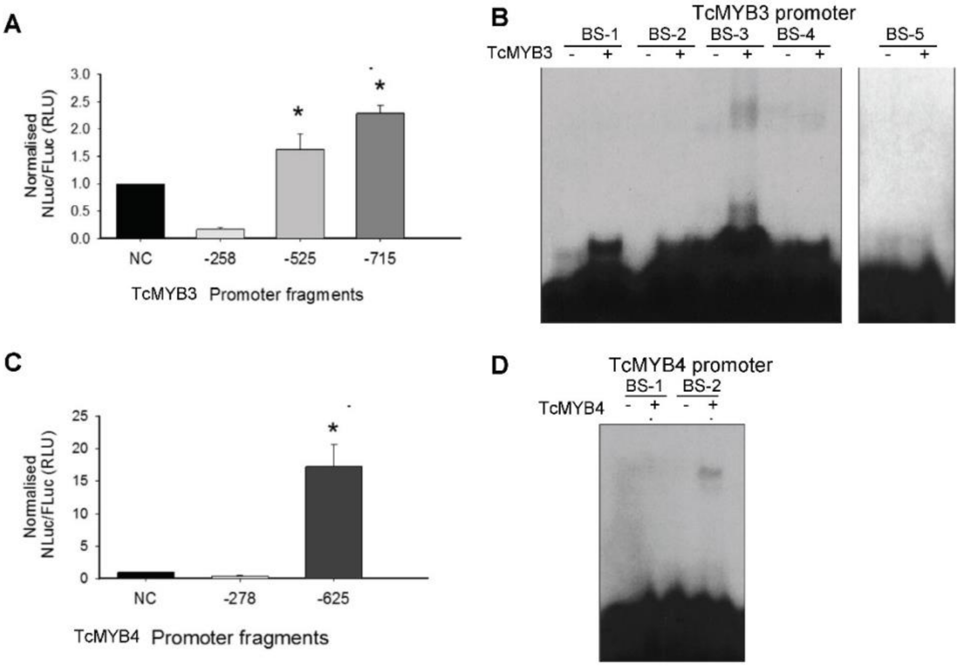

Fig. S8. TcMYB3/4 auto-activation assay.

(A-B) Screening for TcMYB3 binding fragment from *TcMYB3* promoter using TEA in *Taxus* CMC protoplasts (A) and EMSA (B). -715bp: 715 bp before ATG, -525: 525 bp before ATG and -528 bp: 528 bp before ATG. BS site was shown in Fig. 3B. **(C-D)** Screening for TcMYB4 binding fragment from *TcMYB4* promoter using TEA in CMC protoplasts (C) and EMSA (D). -625 bp: 625 bp before ATG, -278: 278 bp before ATG. BS site was shown in Fig3E. The oligonucleotide probe for each binding site (BS) are detailed in Table S4. The values obtained were normalized to the negative control (NC). n >3, significance was determined compared to the NC in each promoter using a student t-test, * p <0.05. Error bars represent standard error.

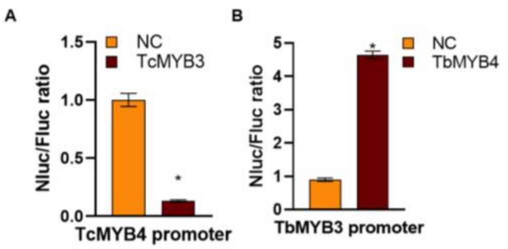

Fig. S9. Trans-activation of TcMYB3 by TcMYB4 in *Taxus* CMCs.

(A-B) Testing the trans-activation of the TcMYB4 promoter by TcMYB3 (A) and trans-activation of the TcMYB3 promoter by TcMYB4 (B) using TEA in CMC protoplasts. Significance was determined compared to the NC in each promoter using a Student's *t*-test, * p value < 0.05, n >3. Error bars represent standard error.

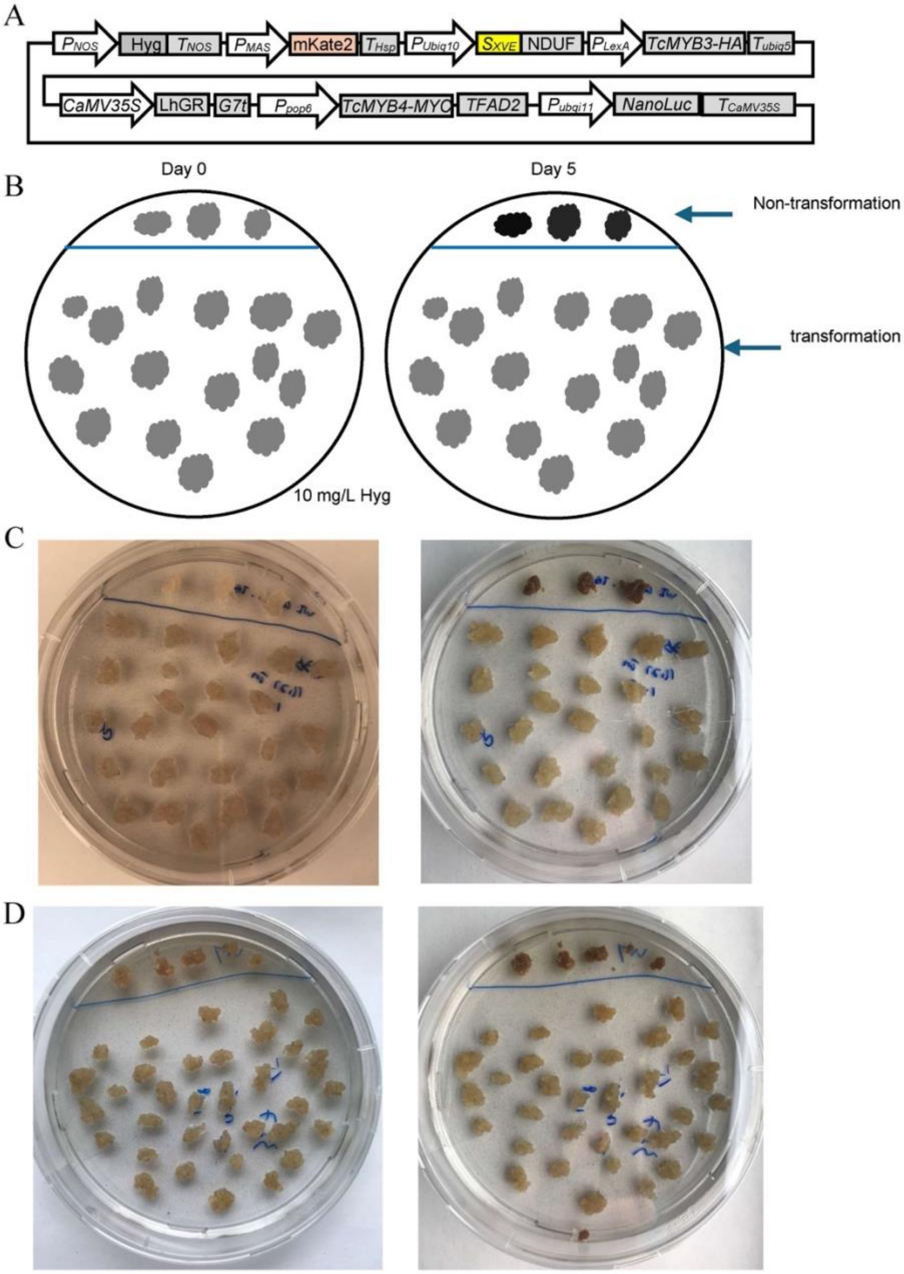

Fig. S10. Generation and selection of transgenic CMC cell lines.

(A) Schematic diagram of the T-DNA region of the binary vector(s) used for CMC transformation. **(B)** Phenotype of wild-type (non-transformed) and transgenic cell lines at day 0 and day 5 post-inoculation on selective medium containing 10 μg L^{-1} hygromycin. **(C-D)** Establishment of stable transgenic CMC cultures. Two independent transgenic lines, designated T1 (Transformation 1) (C) and T2 (Transformation 2) (D), were maintained on selective medium for subsequent metabolic and transcriptional analyses.

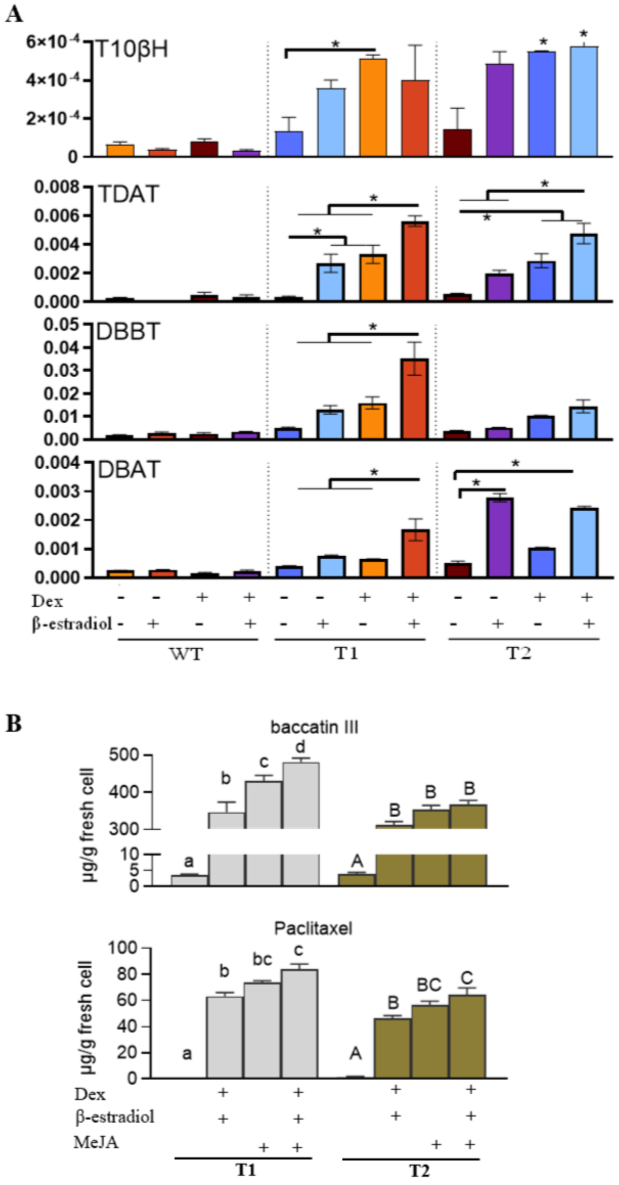

Fig. S11. Molecular engineering Taxus CMCs cells expressing TcMYB3-TcMYB4 module enhance production of paclitaxel, and its key intermediates baccatin III production. (A) Transcriptional expression levels of key genes involved in the biosynthetic pathway of paclitaxel in indicated line following a 12-hour treatment with the indicated chemicals. **(B)** Baccatin III and Paclitaxel yields in μg/g fresh cells following a 7-day treatment with the indicated chemicals. T1 and T2 represent two independent transgenic lines expressing the TcMYB3–TcMYB4 module as shown in Fig. S10. Data are mean ± s.d., n = 3 biological replicates. * in A, and different letters in B indicate significant differences at $P < 0.05$.

Table S1. Primers designed for 5'/3' RACE PCR.

Type	TFs	Gene specific primer 1, 2 & 3 respectively
5' RACE	TcAP2-3	TTCACCCTTGAGGAAGAACG<br>CCGAATTTCAGACACCCATT<br>CCTCTGTCAACTACACAATCCAT
	TcNAC3	CATCTGCCAGACGGTATTCA<br>TGCAAATGGGTTTATCAGTCC<br>CAGATCCACCTCTGCAATGA
	TcbHLH2	GTTGCCTGGAGCTAAGATCG<br>TGATTCAGAGGCTCTTCACG<br>GAGGGCTCTACATCAGAAAGC
3' RACE	TcMYB4	CATCACTGCCGAAGAAGAAC<br>TTTCTAAGTCCACGGCAGAC

Table S2. Primers designed for RT-PCR

TFs	Forward primer	Reverse primer
TcAP2-1	CCTGCTGAATCCGAACATCT	TCACAACCGCTATGGACTTG
TcAP2-2	ACAACTGGAATTGGCCCTCT	CGTTTGCAGAGGAAAGGTGT
TcAP2-3	TCGCAGTCTCGTTGAAGAAA	TCAGTCAGTCCACAGCGAGT
TcAP2-4	GAAGAAAAAGTGCGCAGTCC	TTTGGGCTTCAAATTGCTTC
TcAP2-5	TATTCTTCGCACGCTTCTCC	CGTCTCGGTCTTCTTGGAAA
TcAP2-6	CCAAACCTCTCTGGAAAGCA	GGGCTGAATCCACAACTCAT
TcMYB1	CAACACAACTCGAAGCAGTCA	ACCGTGTAGTCATGGGGTTC
TcMYB2	TTtCCCTCAACAATGGAGGA	cGTTcAcCCCCAGTTTAATG
TcMYB3	TTTCTGGGAGTGGGAACTACA	CCATGAAACAATCCCTCCAT
TcMYB4	CATCCCAATATTCCGACGAC	GGTCTGCCGTGGACTTAGAA
TcNAC1	AATGTTGATGGCGTTCACAG	TGCACGTTGTAGAGGCTGTC
TcNAC2	CAACAACAATGTGCCAACCT	TTGAACGTGAGCTTCTGTGG
TcNAC3	CACAATTGGGATTTGGGTATG	GAAATGCACTGTCGAACACC
TcNAC4	CCCTTTCTCCCAGGTTCTTT	GGGAAGCAAAATGGGTCTAA
TcNAC-5	CGGATTCAGCTCCATTCTTG	ACAGCCAAGGAGCAGGACTA
TcbHLH1	AGCAGTAAGCAACGATGCAG	CTCAAAATGGTTGCCCCTAA
TcbHLH2	GATGGTTTTCTCCATTGTTGC	CATAAGCCTTTGCCTTTTGC
TcWRKY1	CCCATGGATCTGGTTGGTTA	TGTCTTCGACCCGCATAAAT
TcWRKY2	AGGGCTCAGATGGTTACAGC	CAGCCAAGGCTGCAGTATAA
DBTNBT	CCGACAACATTCTTGCATTGAG	CCGTTGCAGGGAACTTACA
T13aH	TCAAGGACGCCGATCAATTC	AATTCCCACCCTGGACAAAC
PAM	TCCAGGGTTCCAAATTCCTTC	AAGAGGACGTGCAGAAAGTG
Actin	GTCCATCCATTGTCCATAGAAA	TGGCAACATTGGTAAAGATATTCA
T5aH	AAAGGGTGGAAGCTGTTGT	GCTTTCCTTCCTGATCGAATCT
BAPT	TAAGCACTCTACAACAACAGG	GCATGAACATTAGTATCTTGATTCC

Table S3. Primers used for cloning of 19 candidate TFs and 10 promoter (pro) for TEA, and recombinant protein expression.

	Forward	Reverse
**Internal control for TEA**		
Rluc SacII		TCCCCGCGGTTATTGTTCATT
Rluc-SpeI	GGACTAGTATGATCCAGAACAAAGGAAACG	
Rluc-attB	GGGGACAAGTTTGTACAAAAAAGCAGGCTTAGAAGGAGATAGAACCATGATCCA GAACAAAGGAAAC	GGGGACCACTTTGTACAAGAAAGCTGGGTCTTATTGTTCATTTTTGAGAACTCG
**TF**		
TcAP2-1	GGGGACAAGTTTGTACAAAAAAGCAGGCTTCATGGCTTCCATGACGAAGGGATTATT	GGGGACCACTTTGTACAAGAAAGCTGGGTCTCAGGCATCCAAGTGTAGGGTGGTG
TcAP2-2	GGGGACAAGTTTGTACAAAAAAGCAGGCTTCATGGAAATATCATGCAAAGGACAGAGCG	GGGGACCACTTTGTACAAGAAAGCTGGGTCTTAGTATTCCTCAAATAGTTCCGACTCCCACA
TcAP2-3	GGGGACAAGTTTGTACAAAAAAGCAGGCTTCATGGCGCGTCGTCGCAGTTGGGTAA	GGGGACCACTTTGTACAAGAAAGCTGGGTCTCAGCCATAGGTCTTCATCCTTAAT
TcAP2-4	GGGGACAAGTTTGTACAAAAAAGCAGGCTTCATGGAGGATCACCAGCATATGAGAG	GGGGACCACTTTGTACAAGAAAGCTGGGTCTCAAAATGTGCTTCTCGTGAAGGAT
TcAP2-5	GGGGACAAGTTTGTACAAAAAAGCAGGCTTCATGGCGTCGACAATGGAAAGGTTAG	GGGGACCACTTTGTACAAGAAAGCTGGGTCCTATTGCCCTTCTTTTTCTTCTTCCTCC
TcAP2-6	GGGGACAAGTTTGTACAAAAAAGCAGGCTTCATGCCGGCCAGGAAGAGGATCCAAA	GGGGACCACTTTGTACAAGAAAGCTGGGTCCTAAGAGAAATGATCAGAAGTAGGAGAAGGG
TcMYB1	GGGGACAAGTTTGTACAAAAAAGCAGGCTTCATGGCTCCAATCCCCAGGCATTCTT	GGGGACCACTTTGTACAAGAAAGCTGGGTCCTATAGACTTTCCCAGAAGTCACATAAATCTG
TcMYB2	GGGGACAAGTTTGTACAAAAAAGCAGGCTTCATGGGGCGAGCTCCGTGCTGTGATA	GGGGACCACTTTGTACAAGAAAGCTGGGTCCTACAACGTAGGCGATGATGCGGCT
TcMYB3	GGGGACAAGTTTGTACAAAAAAGCAGGCTTC ATGTGTCAAACACAAGAGGAAGGAG	GGGGACCACTTTGTACAAGAAAGCTGGGTCTCA ACGAAGTCCTTCTGTGGTACC
TcMYB4	GGGGACAAGTTTGTACAAAAAAGCAGGCTTCATGAGCTCCAAGTGGTTGAATTTGA	GGGGACCACTTTGTACAAGAAAGCTGGGTCTCATATTGGCTGTTGCATTGGAAT
TcNAC1	GGGGACAAGTTTGTACAAAAAAGCAGGCTTCATGGGTAAAGATGGTCTAGAAGGGAAGG	GGGGACCACTTTGTACAAGAAAGCTGGGTCTCACTGGAAAATCATATCAGACGAGTGG
TcNAC2	GGGGACAAGTTTGTACAAAAAAGCAGGCTTCATGAGTGCACATAGTAGAACAAAGACAAC	GGGGACCACTTTGTACAAGAAAGCTGGGTCTCAATTGAGGGTAATACTTGTTGTTGTTGTTGT
TcNAC3	GGGGACAAGTTTGTACAAAAAAGCAGGCTTCATGGGGAGAGAAGGACCTCCC	GGGGACCACTTTGTACAAGAAAGCTGGGTCTTATTGTGGTCTAGGTTGCAGATAG

	GGAC	
TcNAC4	GGGGACAAGTTTGTACAAAAAAGCAGGCTTCATGGAGTCCCTGGCGGTGAATGTGA	GGGGACCACTTTGTACAAGAAAGCTGGGTCTCAATAGCCAGACCACAAACAGTCTGGC
TcNAC5	GGGGACAAGTTTGTACAAAAAAGCAGGCTTCATGTGTCAAACACAAGAGGAAGGAGCC	GGGGACCACTTTGTACAAGAAAGCTGGGTCTCATGTAGTTCCCACTCCCAGAAAATCA
TcbHLH1	GGGGACAAGTTTGTACAAAAAAGCAGGCTTCATGGAGTTGGCAGGTGACCAGGGTT	GGGGACCACTTTGTACAAGAAAGCTGGGTCTTACTCACTCAAAATGGTTGCCCCTAAATTC
TcbHLH2	GGGGACAAGTTTGTACAAAAAAGCAGGCTTCATGAGAACTGAAGATACCCTTCATT	GGGGACCACTTTGTACAAGAAAGCTGGGTCTCATTTAGCAATCACTGTCTGAATC
TcWRKY1	GGGGACAAGTTTGTACAAAAAAGCAGGCTTCATGTTCATGATAAACCTCATG	GGGGACCACTTTGTACAAGAAAGCTGGGTCTTATGAATCTTGAGTCAGAAACCCTTG
TcWRKY2	GGGGACAAGTTTGTACAAAAAAGCAGGCTTCATGGACCAGCACAAAATTGAGCTTTTG	GGGGACCACTTTGTACAAGAAAGCTGGGTCTTATTTTGGGAATAACTCTTGTGTATTTCTGATAAAA
**Promoter**		
TASY*pro*	GGGGACAAGTTTGTACAAAAAAGCAGGCTAACTCGCAATAGCTAGGACATCTT	GGGGACCACTTTGTACAAGAAAGCTGGGTTTCTGCAGAGAGGCAGGG
T5αH*pro*	GGGGACAAGTTTGTACAAAAAAGCAGGCTAGGATCGAGATGTCAAGAA	GGGGACCACTTTGTACAAGAAAGCTGGGTATAATAC TTTCTCGGCCATCA
T13bH*pro*	GGGGACAAGTTTGTACAAAAAAGCAGGCTTATGCCTAAATCTCACGTATGT	GGGGACCACTTTGTACAAGAAAGCTGGGTGGAAAGG AGTAAAGGGGTTA
TDAT*pro*	GGGGACAAGTTTGTACAAAAAAGCAGGCTTGGTGATATATATCAGGATCGAG	GGGGACCACTTTGTACAAGAAAGCTGGGTTGGTCGAAATACTGATAAAAGAG
T10bH*pro*	GGGGACAAGTTTGTACAAAAAAGCAGGCTAACCAG CTGTTATGCCA	GGGGACCACTTTGTACAAGAAAGCTGGGTTTGGAGC AGGTGGGT
DBBT*pro*	GGGGACAAGTTTGTACAAAAAAGCAGGCTCGTCAA ATTTAACATCGCTGTA	GGGGACCACTTTGTACAAGAAAGCTGGGTTGTAGATATTTGGACTCTCTTCTCCT
DBAT*pro*	GGGGACAAGTTTGTACAAAAAAGCAGGCTTCCCACAAAACTAGACAAGTCAT	GGGGACCACTTTGTACAAGAAAGCTGGGTAAAGCTG GGTCTGTTGAGC
PAM*pro*	GGGGACAAGTTTGTACAAAAAAGCAGGCTCTACCTAAACAGACAAGACAACG	GGGGACCACTTTGTACAAGAAAGCTGGGTTGCAGAG CAAGGAAAATAAA
BAPT*pro*	GGGGACAAGTTTGTACAAAAAAGCAGGCTGAATCCCTAACACACACATGCATAC	GGGGACCACTTTGTACAAGAAAGCTGGGTGGAATTGAGCAGCTGAATAATTTTTTAT
DBTNBT*pro*	GGGGACAAGTTTGTACAAAAAAGCAGGCTGTTATA TCGTCGAGGCTTGC	GGGGACCACTTTGTACAAGAAAGCTGGGTCCCAATG ATCCACGAGG

Table S4. Probes used for EMSA

Promoter	Name	Sequence
**TASY**	BS-1-F	AAACACGTGATATGCGCCTG
	BS-1-R	CAGGCGCATATCACGTGTTT
	BS-2-F	AGTAATTACCTAAAATAGAA
	BS-2-R	TTCTATTTTAGGTAATTACT
	BS-3-F	TAAGCAATAACAAAGCACGA
	BS-3-R	TCGTGCTTTGTTATTGCTTA
	BS-4-F	TCAACATATCCATTACATTG
	BS-4-R	CAATGTAATGGATATGTTGA
**T5αH**	BS-1-F	TTCCATGTTTTGTTTTTAGC
	BS-1-R	GCTAAAAACAAAACATGGAA
	BS-2-F	TAATACTAACAAAACGTTGA
	BS-2-R	TCAACGTTTTGTTAGTATTA
	BS-3-F	CTTACGGTTTTGTTTTTGTC
	BS-3-R	GACAAAAACAAAACCGTAAG
	BS-4-F	TGACAACAACTAGAGGGAAG
	BS-4-R	CTTCCCTCTAGTTGTTGTCA
**TDAT**	BS-1-F	CATCCATCCATCCATCATATC
	BS1-R	GATATGATGGATGGATGGATG
	BS-2-F	CTTATTAACTGATAATATGG
	BS-2-R	CCATATTATCAGTTAATAAG
	BS-3-F	ATAAGGAAGATAGCCTTATT
	BS-3-R	AATAAGGCTATCTTCCTTAT
	BS-4-F	TTCAATGTATCCATTGAATT
	BS-4-R	AATTCAATGGATACATTGAA
**T13αH**	BS-1-F	ATTATTCACCAACAATTGTA
	BS-1-R	TACAATTGTTGGTGAATAAT
	BS-2-F	CAACAGATGATAAGGGAGTG
	BS-2-R	CACTCCCTTATCATCTGTTG
	BS-3-F	TGATTTCCCGTTATCAACAG
	BS-3-R	CTGTTGATAACGGGAAATCA
	BS-4-F	CTGAACACGATATAAATGCC
	BS-4-R	GGCATTTATATCGTGTTCAG
**Tβ10H**	BS-1-F	GGCTAAGCACTCTGGAGCGC
	BS-1-R	GCGCTCCAGAGTGCTTAGCC
	BS-2-F	CCAACAAACCAAAAGGGCAT
	BS-2-R	ATGCCCTTTTGGTTTGTTGG
	BS-3-F	CCAGGGGTAGTTTTTCACAT
	BS-3-R	ATGTGAAAAACTACCCCTGG
**DBBT**	BS-1-F	AATTCACAAACCATTTCTGC
	BS-1-R	GCAGAAATGGTTTGTGAATT
	BS-2-F	TTTCTGGACAGTTACATTCT
	BS-2-R	AGAATGTAACTGTCCAGAAA
	BS-3-F	ATCAAGCTAACAGCGACGTA
	BS-3-R	TACGTCGCTGTTAGCTTGAT
	BS-4-F	ACGTATGATATATCTCCACA
	BS-4-R	TGTGGAGATATATCATACGT
**DBAT**	BS-1-F	CGAAGACAAACCATATTTCA

	BS-1-R	TGAAATATGGTTTGTCTTCG
	BS-2-F	AAAAAAAACCAAATAATAAT
	BS-2-R	ATTATTATTTGGTTTTTTTT
	BS-3-F	ATTTCCTTTTAGGTATAAAC
	BS-3-R	GTTTATACCTAAAAGGAAAT
	BS-4-F	TCACATTCTTAGGTGTGTGA
	BS-4-R	TCACACACCTAAGAATGTGA
	BS-5-F	TGTCCTCACCAAAATTAGTT
	BS-5-R	AACTAATTTTGGTGAGGACA
**PAM**	BS-1-F	GACGCAACGATAAATTGCAG
	BS-1-R	CTGCAATTTATCGTTGCGTC
	BS-2-F	CAGCATGTGATAAATGCAAC
	BS-2-R	GTTGCATTTATCACATGCTG
**BAPT**	BS-1-F	TTTCCCTACACAAAAAAGTA
	BS-1-R	TACTTTTTTGTGTAGGGAAA
	BS-2-F	TAGAAAACTGTTAATTAATG
	BS-2-R	CATTAATTAACAGTTTTCTA
	BS-3-F	GATATAGTTTTACATTGGAT
	BS-3-R	ATCCAATGTAAAACTATATC
	BS-4-F	GATTTGTTTCCTTCATTCGA
	BS-4-R	TCGAATGAAGGAAACAAATC
**DBTNBT**	BS-1-F	TCCACTTAGATATTAAAAAA
	BS-1-R	TTTTTTAATATCTAAGTGGA
	BS-2-F	TTAACAAACCAAAACTATAA
	BS-2-R	TTATAGTTTTGGTTTGTTAA
	BS-3-F	AAAAAAATGATATCATTCTT
	BS-3-R	AAGAATGATATCATTTTTTT
	BS-4-F	TTTTTATGGATAGCTGTAAT
	BS-4-R	ATTACAGCTATCCATAAAAA
	BS-5-F	GCCTTTTACCAACCATATTT
	BS-5-R	AAATATGGTTGGTAAAAGGC
**MYB3**	BS-1-F	TTCACTCATTGTTTGGAGTC
	BS-1-R	GACTCCAAACAATGAGTGAA
	BS-2-F	TTTTTCTTGTGTTTTGAGTA
	BS-2-R	TACTCAAAACACAAGAAAAA
	BS-3-F	TCAATGtAGACCACAAAACC
	BS-3-R	GGTTTTGTGGTCTaCATTGA
	BS-4-F	TGTAATGAAACACCCATATG
	BS-4-R	CATATGGGTGTTTCATTACA
	BS-5-F	TGAAATATAAACAATAGAGT
	BS-5-R	ACTCTATTGTTTATATTTCA
**MYB4**	BS-1-F	GTCAACAACGCTCCCTCCCT
	BS-1-R	AGGGAGGGAGCGTTGTTGAC
	BS-2-F	TTTATCTTTGGGTGTGTCTG
	BS-2-R	CAGACACACCCAAAGATAAA

Table S5. TAIL-PCR primers for the promoter region amplification of TcMYB3, TcMYB4.

Name	Orientation	Sequence
TcMYB3-RSP1	R-1	CCCTCTTACTGTTGCTGCTTTCCGAG
TcMYB3-RSP2	R-2	CCAAATTCTGCCCAGTCTGTGCTGG
TcMYB-3-RSP3	R-3	GCTTCCGGTGGCTCCTTCCTCTTG
TcMYB4-RSP1	R-1	CTTGCTAAAGCGCTCCAGCGG
TcMYB4-RSP2	R-2	TATGCAACGAAGAAGACGAATATCCTCC
TcMYB4-RSP3	R-3	CGAATATCCTCCTCGAAAGTCCAGG
AD1	F	NTCGASTWTSGWGTT
AD2	F	NGTCGASWGANAWGAA
AD3	F	WGTGNAGWANCANAGA
AD4	F	AGWGNAGWANCAWAGG
AD5	F	WGCNAGTNAGWANAAG
AD6	F	AWGCANGNCWGANATA
AD7	F	AWGCANGNCWGANATA
